# New insights from monogenic diabetes for “common” type 2 diabetes

**DOI:** 10.3389/fgene.2015.00251

**Published:** 2015-08-07

**Authors:** Divya Sri Priyanka Tallapragada, Seema Bhaskar, Giriraj R. Chandak

**Affiliations:** Genomic Research on Complex Diseases Laboratory, Council of Scientific and Industrial Research-Centre for Cellular and Molecular BiologyHyderabad, India

**Keywords:** simple/Mendelian diseases, complex diseases, type 2 diabetes, monogenic diabetes, maturity onset diabetes of the young

## Abstract

Boundaries between monogenic and complex genetic diseases are becoming increasingly blurred, as a result of better understanding of phenotypes and their genetic determinants. This had a large impact on the way complex disease genetics is now being investigated. Starting with conventional approaches like familial linkage, positional cloning and candidate genes strategies, the scope of complex disease genetics has grown exponentially with scientific and technological advances in recent times. Despite identification of multiple loci harboring common and rare variants associated with complex diseases, interpreting and evaluating their functional role has proven to be difficult. Information from monogenic diseases, especially related to the intermediate traits associated with complex diseases comes handy. The significant overlap between traits and phenotypes of monogenic diseases with related complex diseases provides a platform to understand the disease biology better. In this review, we would discuss about one such complex disease, type 2 diabetes, which shares marked similarity of intermediate traits with different forms of monogenic diabetes.

## Introduction

Genetic diseases have been historically categorized into simple, monogenic and complex, polygenic diseases. This straight forward classification of inherited diseases based on the number of genes involved in precipitating a diseased state proved to be useful in the identification and development of effective diagnostic, genetic counseling strategies, and drug target genes. Studies on single gene- Mendelian disorders have greatly benefitted our understanding of disease causing variants, gene functions and various regulatory mechanisms involved in the normal physiological functioning of the body (Weatherall, 2000). However, this distinction between rare monogenic and common complex diseases is now unclear with blurring boundaries defining these categories (Cooper et al., 2013). One of the best examples driving this point is sickle cell anemia which is caused by single base substitution that replaces glutamic acid with valine in the β-globin (Ingram, 1957). However, marked clinical heterogeneity cannot be explained by this single global mutation but to an extent by different haplotype background in the gamma-globin gene (Steinberg, 2005). Interestingly, the differences among different gamma-globin haplotypes correlate with differences in mean levels of fetal hemoglobin (HbF), which is known to influence the disease severity. Early linkage studies identified *Xmn1*-*HBG2* and *HBS1L-MYB* loci and genome-wide association studies (GWAS) identified polymorphisms at *BCL11A* locus, which predict higher HbF levels and a milder disease course in sickle cell anemia patients (Lettre et al., 2008; Sedgewick et al., 2008; Uda et al., 2008). Together, they account for 20–50% of the variance in HbF levels, illustrating the importance of other genetic loci in modifying disease severity (Menzel and Thein, 2009; Thein et al., 2009). Thus, sickle cell anemia, which was once considered as the simplest of all Mendelian diseases, now strikes as an example of complex disease where multiple factors influence a particular patient's clinical outcome. The story of sickle cell anemia highlighting limitations in the age-old classification of inherited diseases doesn't stand in isolation. This example can now be extrapolated to other Mendelian disorders like phenylketonuria, cystic fibrosis etc. where mutations in one primary gene are present in most of the cases but the phenotype is influenced by allelic heterogeneity and/or mutations at multiple modifier genes (Dipple and McCabe, 2000a).

In today's world, genetic diseases represent a continuum where the phenotype is influenced by the number of loci involved and the extent of environmental participation. This also has direct relation to the methods used for understanding genetic diseases (Dipple and McCabe, 2000b). Techniques utilized for investigating single gene disorders may be applied to identification of genes influencing intermediate traits related to complex diseases. Similarly, methodologies typically used for complex trait analysis like GWAS and next-generation sequencing (NGS) technologies may be applied for identification of modifier genes that influence the phenotypes in monogenic disorders. In this review, we will discuss about one such disease, where defects in single gene are known to cause relatively rare, monogenic forms of diabetes and polymorphisms at several loci are associated with polygenic forms of diabetes. Significant overlap of phenotypes between monogenic diabetes and commonly occurring type 2 diabetes (T2D) provide a great platform to investigate pathophysiological mechanisms underlying development of T2D.

## Diabetes: A complex, chronic metabolic disease

Diabetes is the most prevalent metabolic disease characterized by hyperglycemia due to primary defects in insulin secretion and/or insulin function. Diabetes has reached pandemic proportions with 347 million people affected worldwide which can be traced back to rapid rise in obesity and life style changes like physical inactivity (American Diabetes Association, 2014). Inadequate treatment is potentially devastating due to micro- and macro-vascular complications. Total deaths due to diabetes is expected to rise by 50% in the coming decade projecting it to be the 7th leading global cause of death by 2030 (Mathers and Loncar, 2006). High rate of morbidity and mortality in diabetes is due to the direct and indirect effects of hyperglycemia on the vasculature which include retinopathy causing loss of vision, nephropathy causing renal failure, peripheral neuropathy and autonomic neuropathy and macrovascular complications such as stroke and atherosclerosis (Forbes and Cooper, 2013).

Majority of diabetic cases are broadly classified into two etiological categories: type 1 diabetes (T1D) where lack of insulin secretion due to auto-immune mediated destruction of beta cells causes hyperglycemia and T2D where insulin resistance and inadequate insulin secretory response result in raised circulating glucose levels (American Diabetes Association, 2014). T1D represents around 5–10% (Atkinson et al., 2014) and T2D accounts for about 90% of the diabetic cases worldwide (Wild et al., 2004). Autoimmune destruction of beta cells has multiple genetic predispositions (Onengut-Gumuscu et al., 2015) and effect of environmental factors is still being understood in T1D (Knip et al., 2005; Vaarala et al., 2008). However, markers of immune destruction like islet cell auto-antibodies, auto-antibodies to insulin, GAD65, and tyrosine phosphatases IA-2 and IA-2b and rise in circulating levels of C-peptide help in reasonably accurate diagnosis of T1D and determine their clinical course (Arvan et al., 2012; American Diabetes Association, 2014; Atkinson et al., 2014). In the latter case, decreased insulin secretion and insulin resistance frequently co-exist in patients. T2D, on the other hand is characterized by resistance to insulin action in tissues like liver, skeletal muscle and adipose tissue, which leads to other features such as dyslipidaemia and central obesity (Thomas and Philipson, 2015). Impaired fasting glucose (IFG) or impaired glucose tolerance (IGT) can provide indications of derangements in glucose metabolism (American Diabetes Association, 2014). Patients spend a long asymptomatic period characterized by hyperglycemia, which is undetectable but sufficient to cause pathological changes.

T1D and T2D are polygenic in nature and caused due to interaction between genetic and environmental factors. Monogenic diabetes, on the other hand represents rare, heterogeneous group of disorders due to genetic defects in single genes causing pancreatic beta cell dysfunction and marked hyperglycemia (Thomas and Philipson, 2015). Around 2–5% of global cases correspond to monogenic diabetes. Maturity onset diabetes of the young (MODY) and neonatal diabetes mellitus (NDM) represent two different classes of monogenic diabetes where hyperglycemia is either due to defects in insulin secretion, decrease in beta cell mass or both (Schwitzgebel, 2014). MODY is characterized by features like autosomal dominant inheritance pattern, positive familial history, early age of onset, absence of auto-immune antibodies and insulin resistance (Owen, 2013). Its shared features (Table 1) with other forms of diabetes often results in misdiagnosis of MODY patients as T1D or T2D patients (van der Zwaag et al., 2015). NDM on the other hand is characterized by onset of hyperglycemia in the first few weeks of life and can be either transient neonatal diabetes mellitus (TNDM) or permanent neonatal diabetes mellitus (PNDM). TNDM is a mild form and usually resolves by 18 weeks of age but the patients are at risk of developing diabetes in the adult stages of life where as PNDM requires life-long treatment and can result in isolated hyperglycemia or may present with extra-pancreatic features depending on the gene mutated (Greeley et al., 2011).

**Table 1:** **Characteristic features of different types of diabetes**.

**Features**	**Type 1 diabetes (T1D)**	**Type 2 diabetes (T2D)**	**Maturity onset diabetes of the young (MODY)**
Age of onset	Any age/more frequent childhood and adolescence	More frequent in adults and obese children	Usually before the age of 25 years
Parents affected	Rarely multigenerational	Rarely multigenerational	Usually minimum three generations affected
Inheritance	Polygenic	Polygenic	Monogenic, autosomal dominant/recessive
Beta cell autoantibodies	Present	Absent	Absent
C-peptide	Undetectable/low	Normal/high	Normal
Insulin production	Absent	Present	Present^*^
Obesity	Usually absent	Frequent (>80%)	Very rare
Diabetic ketoacidosis	Common	Rare	Rare
First line treatment	Insulin	Oral hypoglycemic agents (Metformin)	Depends on sub-type of MODY

* *Low levels when pancreatic agenesis occurs due to gene mutations*.

In addition to these categories, few monogenic forms of insulin resistance such as primary lipodystrophic syndromes and insulin receptor defects leading to Donahue syndrome, Rabson–Mendenhall syndrome or Type-A insulin resistance display features of metabolic syndrome and are associated with insulin resistant T2D (Hegele, 2003).

## Genetics of monogenic diabetes: A success story

Linkage studies in families with above diagnostic criteria identified mutations in several genes leading to MODY. Mutations in hepatocyte nuclear factor 4 alpha, (*HNF4A*, MODY1), glucokinase (*GCK*, MODY2), hepatocyte nuclear factor 1 alpha (*HNF1A*, MODY3) result in most common forms of MODY (Shields et al., 2010; Pihoker et al., 2013). Mutations in genes like pancreatic and duodenal homeobox 1 (*PDX1*, MODY4), hepatocyte nuclear factor 1 beta (*HNF1B*, MODY5) neurogenic differentiation 1 (*NEUROD1*, MODY6) and insulin (*INS*, MODY10) cause relatively rare forms of MODY (Naylor and Philipson, 2011). Fine mapping studies and the advent of NGS technologies like whole-exome sequencing, not only rapidly increased the mutational spectrum of causal genes but also helped in the identification of novel MODY genes (Bamshad et al., 2011; Ku et al., 2011; Bonnefond et al., 2012; Johansson et al., 2012). Currently, Online Mendelian Inheritance in Man (OMIM) lists 13 different causal genes that result in distinct sub-phenotypes of MODY as described in Table 2.

**Table 2:** **Genes/loci associated with different forms of monogenic diabetes and insulin resistance**.

**Disease**	**Genes/loci**	**Gene name**	**Chromosome**	**Inheritance pattern**	**Characteristic clinical features**
**MATURITY-ONSET DIABETES OF THE YOUNG (MODY)**					
MODY-1	*HNF4A*	Hepatocyte nuclear factor 4 alpha	20q13.12	AD	Familial, early-onset diabetes. Responds well to sulphonylurea pills
MODY-2	*GCK*	Glucokinase (hexokinase 4)	7p15.3-p15.1	AD/AR	Mild and long-lasting stable hyperglycemia; **mutations can cause PNDM**
MODY-3	*HNF1A*	HNF1 homeobox A	12q24.2	AD	Normoglycemic in childhood, develop progressive beta-cell dysfunction
MODY-4	*PDX1*	Pancreatic and duodenal homeobox 1	13q12.1	AR	Pancreatic agenesis
MODY-5	*HNF1B*	HNF1 homeobox B	17q12	AD/Spontaneous	Exocrine pancreatic insufficiency, renal cysts, genitourinary abnormalities; **mutations can cause TNDM**
MODY-6	*NEUROD1*	Neurogenic differentiation 1	2q32	AR	Very rare, adult onset diabetes with reduced insulin production and associated with obesity; **mutations can cause PNDM**
MODY-7	*KLF11*	Kruppel-like factor 11	2p25	AD	None other than diabetes mellitus
MODY-8	*CEL*	Carboxy ester lipase	9q34.3	AD	Marked exocrine pancreatic is noticed
MODY-9	*PAX4*	Paired box 4	7q32	AD	Adult onset diabetes
MODY-10	*INS*	Insulin	11p15.5	AD	Growth retardation; **mutations can cause PNDM and TNDM**
MODY-11	*BLK*	BLK proto-oncogene, Src family tyrosine kinase	8p23-p22	AD	Adult onset diabetes
MODY-12	*ABCC8*	ATP-binding cassette, sub-family C (CFTR/MRP), member 8	11p15.1	AD	Can cause developmental delay and epilepsy; **mutations can cause PNDM and TNDM**
MODY-13	*KCNJ11*	Potassium channel, inwardly rectifying subfamily J, member 11.	11p15.1	AD	Developmental delay and epilepsy; **mutations can cause PNDM and TNDM**
Mitochondrial mutations	–	Mitochondrial m.3243A>G mutation	–	Mitochondrial	Deafness, short stature, pigmentary retinopathy
**NEONATAL DIABETES MELLITUS (NDM)**					
Permanent neonatal diabetes mellitus (PNDM)	*CISD2*	CDGSH iron sulfur domain 2	4q24	AR	Sensorineural hearing loss, optic atrophy or neuropathy and defective platelet aggregation
	*EIF2AK3/PERK*	Eukaryotic translation initiation factor 2-alpha kinase 3	2p12	AR	Epiphyseal dysplasia, osteoporosis, and growth retardation at later age
	*FOXP3*	Forkhead box P3	Xp11.23	X-linked	Immune dysregulation, polyendocrinopathy, enteropathy, X-linked syndrome
	*GATA6*	GATA binding protein 6	18q11.1-q11.2	AD	Pancreatic agenesis, congenital heart abnormalities
	*GLIS3*	GLIS family zinc finger 3	9p24.2	AR	Congenital hypothyroidism, glaucoma, liver fibrosis and cystic kidney disease
	*IER3IP1*	Immediate early response 3 interacting protein 1	18q12	AR	Microencephaly, infantile seizures
	*MNX1*	Motor neuron and pancreas homeobox 1	7q36	AD	Partial absence of the sacrum, anorectal anomalies and presacral mass
	*NEUROG3*	Neurogenin 3	10q21.3	AR	Diabetes and chronic intractable malabsorptive diarrhea starting soon after birth
	*NKX2-2*	NK2 homeobox 2	20p11.22	NR	Developmental delay with impaired motor and intellectual functionss
	*PCBD1*	Pterin-4 Alpha-CarbinolamineDehydratase/Dimerization Cofactor of HNF-1a	10q22	AR	Early onset diabetes similar to MODY-3; new born screening for phenylketonuria
	*PLAGL1-HYMAI*	Pleiomorphic adenoma gene-like 1; Hydatidiform mole associated and imprinted	6q24	Sporadic	Macroglossia, umbilical hernia, cardiac and brain developmental defects
	*PTF1A*	Pancreas specific transcription factor, 1a	10p12.2	AR	Pancreatic and cerebellar hypoplasia without exocrine dysfunction
	*RFX6*	Regulatory factor X, 6	6q22.1	AR	Intestinal atresia, gall bladder hypoplasia, diarrhea, exocrine pancreatic insufficiency
	*TRMT10A*	TRNA Methyltransferase 10 Homolog A	4q23	NR	Microcephaly with mental retardation, short stature, and early-onset diabetes.
	*WFS1*	Wolframin	4p16.1	AR	Optic atrophy, diabetes insipidus, deafness, renal tract abnormalities, neurological abnormalities
Temporary neonatal diabetes mellitus (TNDM)	*ZPF57*	Zinc finger protein 57	17	AR	Intrauterine growth restriction
	*SLC2A2*	Solute carrier family 2 (facilitated glucose transporter), member 2	3q26.1-q26.2	AR	Hepatomegaly, proximal tubular nephropathy, hypergalactosemia
Permanent/temporary neonatal diabetes mellitus (PNDM/TNDM)	*GATA4*	GATA binding protein 4	8p23.1-p22	AD/De novo	Pancreatic agenesis, congenital heart abnormalities
	*SLC19A2*	Solute carrier family 19 (thiamine transporter), member 2	1q23.3	AR	Megaloblastic anemia, deafness, cardiac and neurological abnormalities
**MONOGENIC FORMS OF INSULIN RESISTANCE**					
CGL type 1	*AGPAT2*	Acylglycerol-3-phosphate O -acyltransferase 2	12q14.1	AR	Very low adiponectinlevels, acanthosisnigricans and hypertrophic cardiomyopathy
CGL type 2	*BSCL2*	Berardinelli-Seip congenital lipodystrophy 2 (Seigin)	11q13	AR	Loss of adipose tissue from mechanical fat pads such as the palms, soles, orbits, scalp and periarticular regions
CGL type 3	*CAV1*	Caveolin-1	7q31.1	AR	Short stature
CGL type 4	*PTRF*	Cavin	17q21.2	AR	Muscular dystrophy
FPLD-type 2 (FPLD2)	*LMNA*	Lamin A	1q22	AD	Preserved/Excess facial and neck fat
FPLD-type 3 (FPLD3)	*PPARG*	Peroxisome proliferator-activated receptor gamma,	3p25	AD	Excess abdominal fat and hypertension
FPLD-type 4 (FPLD4)	*PLIN1*	Perilipin 1	15q26	AD	Loss of subcutaneous adipose tissue primarily affecting lower limbs
Partial lipodystrophy	*TBCD14*	TBC1 domain family, member 4	13q22.2	AD	Acanthosisnigricans and extreme postprandial hyperinsulinemia
	*ZMPSTE24*	Zinc metallopeptidase STE24	1p34	AR	Mandibuloacral dysplasia
	*AKT2*	v-akt murine thymoma viral oncogene homolog 2	19q13.2	AD	Hypoinsulinemic hypoglycemia with hemihypertrophy, dyslipidemia and hepatic steatosis
	*CIDEC*	Cell death-inducing DFFA-like effector c	3p25.3	AR	Preserved facial and neck fat, multiloculated lipid droplets
Leprechaunism (Donahue syndrome), Rabson–Mendenhall syndrome, Type-A insulin resistance	*INSR*	Insulin receptor	19p13.3-p13.2	AD	Acanthosisnigricans, extreme hyperinsulinemia but normal lipid profile, preserved adiponectin levels

*AD, Autosomal dominant; AR, Autosomal recessive; PNDM, permanent neonatal diabetes mellitus; TNDM, temporary neonatal diabetes mellitus; CGL, Congenital Lipodystrophy (Berardinelli-Seip syndrome); FPLD, Familial partial lipodystrophy*.

TNDM is most commonly caused due to over-expression of imprinted genes, *PLAGL1* and *HYMAI* at 6q24 due to paternal uniparental disomy of chromosome 6 or duplication of 6q24 on the paternally inherited allele or hypomethylation of the maternally inherited 6q24 allele (Temple and Shield, 2002). Other rarer causes of TNDM include heterozygous mutations in genes encoding sub-units of ATP-sensitive potassium channel: potassium channel, inwardly rectifying subfamily J, member 11 (*KCNJ11*) (Flanagan et al., 2007), ATP-binding cassette, sub-family C, member 8 (*ABCC8*) (Patch et al., 2007) and bi-allelic mutations in zinc finger protein 57 (*ZFP57*) (Mackay et al., 2008). In addition, rare mutations in *INS* may also cause a phenotype which can be described as TNDM (Stoy et al., 2007). More than 50% of PNDM cases are due to activating mutations in *KCNJ11* and *ABCC8*, while majority of mutations genes associated with PNDM are de novo in origin (Gloyn et al., 2004; Babenko et al., 2006; Edghill et al., 2010). Approximately 20% of PNDM is attributed to mutations of *INS* (Stoy et al., 2007). Rarely PNDM is caused by inactivating mutations of *PDX1* and glucose transporter2 (*SLC2A2*) (Nicolino et al., 2010). As of date, mutations in close to 20 genes are known to cause diabetes in the first year of life (Table 2).

Hyperglycemia is a common consequence of mutations in these genes; nevertheless, they are associated with distinct phenotype and characteristic clinical presentation. For instance, mutations in *HNF4A* are associated with increase in birth weight, macrosomia, hypoglycemia at birth and adolescent onset of diabetes (Pearson et al., 2007). Patients exhibit progressive beta cell failure and long term treatment with low dose sulphonylureas is proven to be more effective than insulin treatment (Fajans and Brown, 1993). In addition to the above features, patients carrying *HNF-1A* mutations present with severe dysfunction of hepatocytes and renal tubular cells resulting in glycosuria with high incidence of vascular complications. Patients with *HNF1A* mutations are also extremely sensitive to low doses of sulphonylureas (Pearson et al., 2000). Mutations in *HNF1B* are associated with renal defects with cystic renal disease, pancreatic agenesis and genitor-urinary abnormalities. But these patients are insensitive to treatment with insulin sensitizers and require early treatment with insulin (Edghill et al., 2006). In contrast to the mutations in various *HNF* genes, glucokinase mutations result in mild, stable, fasting hyperglycemia from birth and small elevations in post-prandial glucose levels. Insulin secretion remains intact and vascular complications are not commonly observed in these patients and hence they do not require any pharmacological intervention and dietary intervention is usually sufficient to maintain stable glycosylated hemoglobin levels (HbA1c) levels (Stride et al., 2002; Murphy et al., 2008). Activating *KCNJ11* mutations are associated with a number of clinical features ranging from isolated diabetes to more severe phenotypes such as developmental delay-epilepsy-neonatal diabetes syndrome (Gloyn et al., 2006). Neonatal patients identified with *KCNJ11* and *ABCC8* mutations show little endogenous insulin secretion and non-detectable C-peptide levels and hence used to be put on long-term insulin treatment. However, understanding of mechanism of action of sulphonylureas led to shift of therapeutic regimen from insulin injections to high sulphonylureas with improved glycemic control, lower costs of treatment and better quality of life (Sagen et al., 2004; Pearson et al., 2006; Mlynarski et al., 2007; Rafiq et al., 2008). This stands as a classical example of application of genetic information for personalized medicine.

The success in the field of monogenic diabetes can largely be attributed to a distinct phenotype associated with each sub-class, and strong genotype-phenotype correlation that allowed room for better diagnostic- and genotype- based management options. Genetic dissection of different forms of monogenic diabetes sheds light on beta cell physiology and improved understanding of insulin secretion and its regulation. With rapid advances and decreased cost of the NGS technologies, it is expected that screening of people affected with monogenic diabetes would unravel novel genetic etiologies of pancreatic beta cell dysfunction (Bamshad et al., 2011; Ellard et al., 2013).

## Type 2 diabetes is a syndrome, not a single disease

From the above section, it is evident that monogenic diabetes has a clear genetic basis and a reasonably distinct phenotypic presentation for each sub-class. T1D also has clear autoimmune origin leading to absolute insulin deficiency in vast proportion of cases. These facts have guided benefits of early intensive glycemic control (HbA1c below 6%) compared to conventional approach (HbA1c between 7 and 7.9%) in T1D patients as demonstrated in Diabetes Control and Complications Trial (DCCT) and follow-up Epidemiology of Diabetes Interventions and Complications (EDIC) clinical trials which showed significant reduction in the risk of developing both micro and macrovascular complications associated with chronic hyperglycemia (Diabetes Control and Complications Trial, 2005).

In a sharp contrast, T2D does not have a known directly identifiable cause. It is characterized by progressive insulin resistance in insulin responsive tissues, dyslipidaemia, abdominal obesity, and rise in pro-coagulant and pro-inflammatory factors (Thomas and Philipson, 2015). Multitude of studies have revealed the confounding effects of risk factors like age, sex, physical inactivity, smoking, diet, ethnicity, family history, and gestation. Adding to the complexity, chronic exposure to stress, low socio-economic status and psycho-social factors are also shown to be risk factors for T2D from recent environment-wide association studies. The diagnosis of T2D can rather be considered “*waste basket diagnosis”*—not because there is *no-cause*, but because there is *no-one-cause*. The rate of disease progression and advent of long-term complications is not similar in all patients and hence treatment regimen needs individual tailoring based on suitable assessment of risk factors involved. While follow-up of United Kingdom Prospective Diabetes Study (UKPDS) showed that tight glycemic control decreases macrovascular end points in patients with T2D (Group UKPDS, 1998), other studies like Action to Control Cardiovascular Risk in Diabetes (ACCORD) and Action in Diabetes and Vascular Disease (ADVANCE) trials did not provide consistent results. Type 2 diabetics with intensive blood glucose-lowering treatment arm had an increased risk of cardiovascular death in ACCORD trial compared to those in the conventional treatment arm (Ismail-Beigi et al., 2011). Although the ADVANCE trial did not exhibit increased mortality, it did not reveal any significant benefit on macrovascular outcome (Zoungas et al., 2009). All these observations strongly indicate that T2D is not a single disease, but, a conglomeration of metabolic disorders characterized by a combination of multiple intermediate traits like increased IFG, IGT, body mass index (BMI), waist-hip-ratio (WHR), dyslipidemia and hypertension. This very reason led to a paradigm shift in the field of genetics of T2D, which is discussed in detail in the next section.

## Genetics of type 2 diabetes: An unexpected failure or a missed opportunity

Several lines of evidence support the principle of inherited genetic susceptibility as an important risk factor for T2D. Offspring of a parent with T2D face a 40% lifetime risk of developing T2D, which increases to 70% when both parents have T2D. High concordance rate obtained in monozygotic twins (96%) suggests a strong genetic component of this multi-factorial disease (Barnett et al., 1981). Furthermore, 40% of first-degree relatives of T2D patients develop diabetes as compared to 6% in the general population (Kobberling and Tillil, 1982). Its substantial genetic component propelled geneticists from all over the globe to devote huge efforts for the hunt of T2D susceptibility genes.

Initial attempts were through genome-wide familial linkage-based approaches using multi-generational pedigrees and/or affected sib-pairs studies where the analysis relies on shared chromosomal regions inherited from common ancestors to identify familial genetic variants with large consequences. While this method largely helped in genetic studies of monogenic diabetes, its application to common T2D has been a limited success. Several chromosomal regions showed evidence of linkage, but positional cloning of the putative causative genes has not been successful for most of the regions except few like *HNF4A*, calpain 10 (*CAPN10*), ectonucleotidepyrophosphatase/phosphodiesterase 1 (*ENPP1*), adiponectin, C1Q and collagen domain containing (*ADIPOQ*) and transcription factor 7 like 2 (*TCF7L2*) (Florez et al., 2003). Parallel to linkage studies, candidate gene approach was employed by researchers to identify T2D risk associated genes. Selection of candidate genes was typically based on hypothesis about biological mechanisms that are putatively involved in T2D pathogenesis, hence, it was plausible to conceive that variants in these genes could predispose to disease or related phenotype. Genes influencing pancreatic beta cell function, like *ABCC8, KCNJ11, SLC2A2, HNF4A, INS*, and genes influencing insulin action like *PPARG, INSR, PIK3R1, IRS1, IRS2*, and *SOS1* were among the initially identified candidate genes that significantly alter disease susceptibility (McCarthy, 2004). Despite many positive reports, associations could be replicated rarely in other studies mainly because of the small sample sizes used in these studies. Furthermore, poor functional characterization of much of the genome made it impossible to make fully informed decisions while selecting candidate genes for association studies.

At this instance, completion of the Human Genome and International HapMap Projects came as a major boon to the then growing field of T2D genetics. Identifying genetic associations in a genomic fashion i.e., GWAS became a reality. Based on the “common variant- common disease (CVCD) hypothesis” that the genetic risk for common complex diseases is often due to disease producing alleles found at relatively high frequencies (>1%), GWA studies were designed to pick up multiple common variants associated with disease risk (Reich and Lander, 2001). The first wave of GWAS was conducted in Europeans and identified a dozen loci, of which 8 T2D susceptibility loci have been replicated across multiple ethnic groups: *TCF7L2, SLC30A8, HHEX, CDKAL1, IGF2BP2, CDKN2A*/*B, PPARG*, and *KCNJ11* (Cauchi et al., 2007; Lee et al., 2008; Ng et al., 2008; Wu et al., 2008; Hu et al., 2009; Rong et al., 2009; Chauhan et al., 2010; Han et al., 2010). In parallel, strong association between *FTO* variants and T2D was replicated in three independent studies, but the association signal weakened after adjustment for BMI, suggesting that variants mediate risk partly through increased adiposity (Dina et al., 2007; Frayling et al., 2007; Scuteri et al., 2007). While the first wave of studies employed logistic analyses and investigated T2D as a dichotomous phenotype in cases and controls, the second wave of GWAS loci were identified from linear analysis of continuous glycemic traits and anthropometric traits in participants without diabetes. Different consortia were formed to increase the sample size, which facilitated detection of low-effect common variants associated with T2D risk. One of the early consortiums was the DIAbetes Genetics Replication And Meta-analysis consortium (DIAGRAM) aimed at characterizing genetic basis of T2D, mainly in individuals of European descent and identified 31 novel loci associated with increased T2D risk (Saxena et al., 2007; Sladek et al., 2007; Zeggini et al., 2008; Voight et al., 2010; Morris et al., 2012; Scott et al., 2012). Another major collaborative effort to dissect loci that impact glycemic and metabolic traits was the Meta-Analyses of Glucose and Insulin-related traits Consortium (MAGIC). Several loci influencing traits like fasting plasma glucose (FPG), fasting insulin, 2h glucose and HbA1c were identified and many of them like *ADCY5, PROX1, GCK, GCKR*, and *DGKB-TMEM195* also influenced risk of T2D (Dupuis et al., 2010; Soranzo et al., 2010; Strawbridge et al., 2010; Manning et al., 2012; Scott et al., 2012). The Genetic Investigation of ANthropometric Traits (GIANT) consortium dedicated efforts to identify genetic loci that modulate obesity related traits like height, BMI, WHR, WHR adjusted for BMI and waist and hip circumference. So far, common genetic variants at hundreds of loci associated with above anthropometric traits have been identified (Berndt et al., 2013; Randall et al., 2013; Locke et al., 2015; Shungin et al., 2015).

In parallel to these studies, GWAS and meta-analyses of GWAS performed in other ethnicities (i.e., East Asians, South Asians, and African Americans) revealed multiple new T2D-associated genes (Billings and Florez, 2010). For example, six novel signals *GRB14, ST6GAL1, VPS26A, HMG20A, AP3S2*, and *HNF4A* loci were identified from a meta-analysis of GWAS in South Asian population (Kooner et al., 2011). Interestingly, many, but not all of the variants identified in Europeans have been replicated in non-Europeans and amongst the replicated loci, differences in allelic frequencies and effect sizes have been observed (Yajnik et al., 2009). Put together, these results highlight similarity in genetic susceptibility as well as ethnic differences indicating that genetic variability strongly influences magnitude of the effect of GWAS risk loci in different populations. Adding to the complexity, amongst close to 80 loci associated with T2D risk (Data Sheet 3 in Supplementary Material), other than *TCF7L2*, there is absence of any large single-gene effects (Billings and Florez, 2010). Together these loci account for less than 20% of the T2D heritability due to their small effect sizes and point to reasons for the “missing heritability” in T2D (Manolio et al., 2009). Furthermore, resolving functional mechanism of the genetic association and establishing causality has proven to be extremely challenging, primarily because of the fact that large percentage of associations are determined by non-coding variants. This hugely supported the notion that high-penetrant rare-variants (minor allele frequency < 1%) could be the primary players in common diseases and may have a strong impact on disease risk (Pritchard and Cox, 2002; Carlson et al., 2004).

While GWAS have implicated previously unknown genes and pathways in T2D pathogenesis, next generation sequencing studies like whole genome and exome sequencing aimed at identifying variants of low frequency with large effect size and likely elucidate disease causing mechanisms (Cirulli and Goldstein, 2010; Steinthorsdottir et al., 2014). Whole genome sequencing of Icelanders has identified low-frequency and rare sequence variants in *CCDN2, PAM* and *PDX1* that are associated with altered T2D risk (Flannick et al., 2014). Similarly, genotyping of ~150,000 individuals across 5 ancestry groups has identified 12 loss of function mutations in *SLC30A8* which decrease risk of T2D (Majithia et al., 2014). Exome sequencing of *PPARG* in 19,752 T2D cases and controls from multiple studies and ethnic groups identified rare variants which are associated with decreased activity in adipocyte differentiation and increased T2D risk (Claussnitzer et al., 2014). While these technologies identify additional genetic changes that contribute to risk of T2D, obtaining a huge sample size for the detection of low frequency variants remains a huge challenge.

## MODY and T2D: Common loci-common mechanisms

The GWAS and post-GWAS era witnessed tremendous advancements made on the technological front and parallel developments in statistical methodology and inference, analytical frameworks, and computational tools, yet translation of the research findings for accurate risk prediction and subsequent disease prevention, discovery of new drug targets, personalization of medicine remains an unsolved challenge. Increasing efforts are now being devoted to understand the functional mechanisms underlying genetic association of loci with T2D risk and associated quantitative glycemic and/or anthropometric traits. Owing to the large number of associations found with genetic variants in the non-protein coding region or gene desert regions, special focus is laid on decoding the nature of these non-coding variants (Fogarty et al., 2014). Recent fine mapping and functional characterization of GWAS variants provide several evidences where *cis*-regulation is a common mechanism underlying these associations and most frequently affected elements include transcriptional enhancers and silencers (Winckler et al., 2007; Fogarty et al., 2013, 2014). Thus, identification of target genes of *cis*-regulatory variants is necessary to understand the functional mechanisms by which these variants act.

As these efforts continue, gaining novel insights from the molecular genetics of monogenic forms of diabetes would help propel research in the field of common T2D genetics toward a translational level. From the research efforts in identifying genes responsible for monogenic forms of diabetes and findings from GWAS for T2D, strong evidence has emerged that several loci implicated in monogenic diabetes are also involved in mediating risk of polygenic T2D. As a matter of fact, most of the T2D susceptibility genes identified in the pre-GWAS era are key players in the development of some forms of monogenic diabetes; *KCNJ11, HNF4A, SLC2A2, PPARG*, and *INSR*. Recent studies identified few other monogenic diabetes loci like *HNF1A, HNF1B, GCK, PDX1, GLIS3, WFS1, PAX4*, and *LMNA* which harbor variants associated with T2D risk (Sidransky, 2006; Sandhu et al., 2007; Wegner et al., 2007; Voight et al., 2010; Rees et al., 2011; Cho et al., 2012). Interestingly, several variants in these loci influence intermediate glycemic and anthropometric traits associated with T2D like FPG, insulin related traits, lipid related traits, Hb1Ac levels, C-reactive protein, homocysteine and waist circumference. Complete list of loci common between T2D and monogenic diabetes and associated phenotypes is presented in Table 3.

**Table 3:** **Genes/loci common between monogenic diabetes and type 2 diabetes and related intermediate traits**.

**Genes/loci**	**Trait**	**SNP(s)**	**Nature of variant**	**PubMed IDs**
*GLIS3*	FPG and FPG levels adjusted for BMI	rs7034200	Intronic	22581228, 20081858
	HOMA-B	rs7034200	Intronic	20081858
*HNF1A*	Cholesterol, total	rs1169288, rs1800961	Missense	20686565
	LDL cholesterol	rs1169288, rs2650000	Missense	24097068, 19060906
	Homocysteine levels	rs2251468	Intronic	23824729
	C-reactive protein	rs1183910, rs7979473, rs7310409, rs2393791, rs2259816, rs7305618	Intronic	23844046, 22939635, 21647738, 21196492, 19567438, 18439548, 24763700
		rs1169310	3′ UTR	18439552
*HNF4A*	Cholesterol, total	rs1800961	Missense	24097068, 20686565
	HDL cholesterol	rs1800961	Missense	24097068, 19060906, 24097068
	C-reactive protein	rs1800961	Missense	21300955, 22939635
*GCK*	FPG	rs4607517, rs1799884, rs3757840	Intronic	19060907, 20081858, 23575436, 22399527
	FPG adjusted for BMI	rs4607517, rs2293941	Intronic	22581228
	1-h plasma glucose and 2-h plasma glucose	rs1799884	Intronic	23575436
	Glycated hemoglobin levels	rs1799884, rs730497, rs4607517	Intronic	24647736, 24244560, 20858683, 19096518
	HOMA-B	rs4607517	Intronic	20081858
*PPARG*	Fasting insulin adjusted for BMI	rs1801282	Missense	22581228
	WHR adjusted for BMI in women	rs4684854	Intergenic	23754948
*LMNA*	Cholesterol, total	rs577492	Intronic	17327437
	Waist circumference	rs11578696, rs955383	Intronic	17327437
*SLC2A2*	FPG and FPG levels adjusted for BMI	rs11920090	Intronic	20081858, 22581228
	HOMA-B	rs11920090	Intronic	20081858
*INSR*	Triglycerides	rs7248104	Intronic	24097068

*SNP, single nucleotide polymorphism; FPG, Fasting plasma glucose; PG, plasma glucose; BMI, body mass index; HOMA-B, homeostasis model assessment of beta cell function; LDL, low density lipoprotein; HDL, high density lipoprotein; UTR, untranslated region; WHR, waist-hip ratio*.

It is interesting to observe that loci common/shared between monogenic diabetes and T2D affect physiological processes like pancreatic beta cell development and function, glucose sensing mechanisms and their coupling with insulin release/secretion and endoplasmic reticulum (ER) stress. Most of the common loci are transcription factors, which play key role in beta cell development. For example, *PDX1* directs the early embryonic development of pancreas and differentiation of insulin-producing islet β cells in endocrine pancreas by forming cross-regulatory transcriptional networks where *FOXA2* and *HNF1B*, other T2D associated genes are also key players (Oliver-Krasinski et al., 2009; Arda et al., 2013). It also promotes expression of *INS, SLC2A2*, and *GCK*, which are essential for insulin synthesis and glucose sensing. HNF1A also controls beta cell function by regulating target genes like *SLC2A2, HNF4A*, pyruvate kinase, and hepatocyte growth factor activator (Servitja et al., 2009). Genes encoding ATP-sensitive potassium channel sub-units *KCNJ11* and *ABCC8* couple cell metabolism with membrane potential and play a central role in regulation of insulin secretion in pancreatic beta cells. Mutations in these genes form high-risk haplotype which affect progression from IGT to T2D (Laukkanen et al., 2004). *WFS1* forms component of unfolded protein response and maintains homeostasis of the ER in pancreatic beta cells. Inactivating mutations in *WFS1* result in ER stress causing beta cell dysfunction and recently *HNF4A* is also shown to regulate beta cell susceptibility to ER stress (Fonseca et al., 2005; Takei et al., 2006).

Deciphering a role for genes implicated in monogenic diabetes to T2D has the advantage of carrying the benefits along with. For example, *GCK* gene product, hexokinase 4 catalyzes the first step of glycolysis and is expressed in both hepatocytes and pancreatic beta cells. The beta cell isoform is a glucose sensor and plays an important role in glucose stimulated insulin secretion (Matschinsky, 1990, 2002). Common variants in *GCK* predict T2D risk by influencing various T2D intermediate phenotypes like FPG, HbA1c levels, and HOMA-B (Dupuis et al., 2010; Soranzo et al., 2010; Manning et al., 2012; Chen et al., 2013). A recent study has revealed that patients harboring *GCK* mutations reportedly have a low prevalence of vascular complications highlighting the probable limited role of isolated, mild hyperglycemia in mediating diabetic vascular complications (Steele et al., 2014). In this scenario, it may not be unreasonable to surmise a possible role for variants in *GCK* or other monogenic diabetes associated genes modifying the T2D disease course in presence of other precipitating factors. However, it is important to note that these common loci are causal in case of monogenic diabetes, yet they contribute merely to T2D pathogenesis and this discrepancy is largely explained by mutational heterogeneity and the nature of variants at these loci (Sidransky, 2006). While mostly, coding and rare variants cause monogenic diabetes, non-coding and regulatory variants at the same loci are associated with T2D and related intermediate traits. This, in turn, also accounts for differences in the effect sizes of associated variants and phenotypic heterogeneity between monogenic diabetes and T2D. Regulatory nature of variants associated with T2D and intermediate traits also entails gene-gene and gene-environment interactions, of which the later mainly results in modulation of variant effects with various factors like physical activity, nutrition, stress, etc. (Buil et al., 2015). Thus, in contrast to the monogenic diabetes, screening for the common variants in these loci to decide the clinical course of T2D is still a long sought dream.

## Role of environmental factors: Epigenetic regulation of candidate genes

Epigenetic mechanisms like DNA methylation, covalent histone modifications, non-coding RNAs, microRNAs are plausible means by which environmental factors integrate their effects with genetic variants to mediate T2D risk (Ling and Groop, 2009; Waki et al., 2012). Of all, the most thoroughly studied epigenetic modification is the methylation of cytosine-phosphatidyl-guanine (CpG) sites which is associated with post-translational histone modifications and results in altered chromatin structure and consequent differences in gene expression (Jaenisch and Bird, 2003). The loci shared between monogenic diabetes and T2D serve as good example to study these interactions. Mutations in transcription factors like *HNF1A, HNF1B, HNF4A, PDX1*, and *NEUROD1* modulate their affinity for both histone modifying enzymes and DNA leading to disruption of regulatory interactions (Ling and Groop, 2009). Several T2D associated variants either introduce or remove a CpG site, which results in differential DNA methylation and altered expression of associated or near-by genes. Tissue specific DNA methylation of common loci like *PPARG, WFS1, IRS1, PDX1*, and *INS* have been reported in independent studies (Dayeh et al., 2013; Nilsson et al., 2014). For example, differential methylation has been observed in common loci like *PPARG* and *IRS1* in adipose tissue from unrelated T2D patients compared with control individuals and expression of *PDX1* and *INS* was decreased in pancreatic islets from diabetics compared to non-diabetic donors (Yang et al., 2011, 2012). Interestingly, quantitative DNA methylation analysis also shows consistent differences in DNA methylation patterns between populations of different ethnicities at common loci *KCNJ11* along with other loci like *ADCY5* and *FTO* which are attributed to genetic and/or region-specific environmental factors (Elliott et al., 2013).

In addition, epigenetic modifications are proposed as key mechanisms in mediating effect of nutritional disturbances that result in fetal programming and/or metabolic programming for future risk of cardio-metabolic disorders and is current topic of scientific probity. The fetal environment is influenced by several factors and maternal under and/or over nutrition, and interaction between fetal and maternal environment were found as major determinants mediating metabolic programming (Aagaard-Tillery et al., 2008; Yajnik and Deshmukh, 2012). Several epidemiological and animal studies provide evidence for impact of suboptimal uterine environment and early neonatal life on the offspring susceptibility to chronic diseases like T2D in adult life (Snoeck et al., 1990; Breier et al., 2001; Ozanne and Hales, 2002). Epigenetic mechanisms, especially changes in DNA methylation have been shown to modulate effect of these determinants on the altered expression of key genes involved in fetal metabolism and development (Burdge et al., 2007; Gonzalez-Bulnes and Ovilo, 2011). The critical issue is that fetal programming is even transmitted to the next generations highlighting the possibility of transgenerational epigenetic inheritance of disease susceptibility (Waki et al., 2012). Studies on human cord blood samples from neonates with intrauterine growth restriction due to maternal low protein diet has shown dysregulation of *HNF4A* methylation and other loci encoding *HNF4A*-interacting proteins (Sandovici et al., 2011). Similarly, maternal and paternal high fat diet consumption is associated with global methylation changes and induces glucose intolerance and insulin resistance in offspring (Aagaard-Tillery et al., 2008; Gallou-Kabani et al., 2010; Ng et al., 2010). Effect of maternal micronutrients like folic acid (FA) and vitamin B12 (B12) on fetal development and increased obesity risk is already established (Yajnik et al., 2008). Maternal micronutrient deficiency (FA and vitamin B12) results in global methylation changes in livers of pups leading to decrease in expression of *PPAR*α and *PPAR*γ genes and supplementation of omega-3 fatty acid to these pups reduced the global DNA methylation and restored the expression of these transcription factors highlighting the role of poly unsaturated fatty acids and their interaction with one-carbon metabolism (Kulkarni et al., 2011). All the above results indicate strong role of epigenetic modifications not only in mediating the effects of common variants in shared loci but also in programming fetus for future risk of metabolic disturbances.

## Future perspectives

Developments in the field of T2D genetics have undoubtedly led to identification of new loci like *GLIS3* (Cho et al., 2012), *PAX4* (Ma et al., 2013), and *GCK* (Muller et al., 2014) that are common between monogenic diabetes and T2D. However, expanding mutational spectrum of these common loci using techniques like whole exome sequencing has now reached a point of saturation. At this point, understanding intricate mechanisms of interaction of these common variants with environmental factors in manifesting a syndrome like T2D would help us expand basis of phenotypic heterogeneity in the background of mutational heterogeneity. A recent study provides experimental evidence for a new functional approach to investigate epigenetics of T2D (Fogarty et al., 2014). Epigenetic conservation of dysregulated loci in high fat diet fed mice and obese humans as observed in this study substantiates usage of animal models to understand effect of various environmental factors in modifying disease risk. Moreover, overlap between epigenetically regulated regions with nominally associated T2D risk loci suggests that present approach can complement human genetic studies to assess clinical risk of such loci. Besides mediating epigenetic changes, variants in these common loci also explain significant proportion of variance in response to treatment options currently available for T2D. *KCNJ11* and *PPARG* variants provide evidence of a successful pharmacogenetic approach for treatment of T2D (Gloyn et al., 2001; Bluher et al., 2003). Several common polymorphisms in genes like *KCNJ11, ABCC8, WFS1, GLIS3, HNF4A, HNF1B* and *GCK* that have common antecedents in monogenetic diabetes and T2D have influence on the glycemic outcomes of metformin treatment (Jablonski et al., 2010). Common variants in *KCNJ11, ABCC8, NEUROD1* and *PAX4* predict response to repaglinide for glycemic outcomes like glycated hemoglobin, fasting glucose and post-prandial glucose (He et al., 2008; Florez et al., 2012; Gong et al., 2012). Though, these examples foster the potential for utilization of these variants in common loci to personalize medicine, understanding diversity of drug responders, metabolic and signaling pathways associated with a drug's action supported by clinical observations is required for tailoring therapeutic needs. In addition, exploring new avenues like brain centered glucose regulation and investigating the role of common loci in central regulation of glucose metabolism would help us expand our horizons and improve our understanding of disease biology.

## Conclusions

To conclude, no genetic disease is monogenetic now, thanks to better understanding of the clinical phenotype and to the various technological advances that allow the whole “OME” to be investigated. Understanding and utilization of single gene effects on specific traits that conglomerate into a complex phenotype is currently the best way to understand the genetic basis at functional level and be of any use for disease prevention and management. However, lot of factors such as mutational heterogeneity, nature of variants, ethnic differences etc. need to be understood before this becomes a practical reality.

### Conflict of interest statement

The authors declare that the research was conducted in the absence of any commercial or financial relationships that could be construed as a potential conflict of interest.

## References

[B1] Aagaard-Tillery K. M. Grove K. Bishop J. Ke X. Fu Q. McKnight R.. (2008). Developmental origins of disease and determinants of chromatin structure: maternal diet modifies the primate fetal epigenome. J. Mol. Endocrinol. 41, 91–102. 10.1677/JME-08-0025 18515302 PMC2959100

[B2] American Diabetes Association. (2014). Diagnosis and classification of diabetes mellitus. Diabetes Care 33, S62–S69. 10.2337/dc14-s081 21193628 PMC3006051

[B3] Arda H. E. Benitez C. M. Kim S. K. (2013). Gene regulatory networks governing pancreas development. Dev. Cell 25, 5–13. 10.1016/j.devcel.2013.03.016 23597482 PMC3645877

[B4] Arvan P. Pietropaolo M. Ostrov D. Rhodes C. J. (2012). Islet autoantigens: structure, function, localization, and regulation. Cold Spring Harb. Perspect. Med. 2:a007658. 10.1101/cshperspect.a007658 22908193 PMC3405822

[B5] Atkinson M. A. Eisenbarth G. S. Michels A. W. (2014). Type 1 diabetes. Lancet 383, 69–82. 10.1016/S0140-6736(13)60591-7 23890997 PMC4380133

[B6] Babenko A. P. Polak M. Cave H. Busiah K. Czernichow P. Scharfmann R.. (2006). Activating mutations in the ABCC8 gene in neonatal diabetes mellitus. N. Engl. J. Med. 355, 456–466. 10.1056/NEJMoa055068 16885549

[B7] Bamshad M. J. Ng S. B. Bigham A. W. Tabor H. K. Emond M. J. Nickerson D. A.. (2011). Exome sequencing as a tool for Mendelian disease gene discovery. Nat. Rev. Genet. 12, 745–755. 10.1038/nrg3031 21946919

[B8] Barnett A. H. Eff C. Leslie R. D. Pyke D. A. (1981). Diabetes in identical twins. Diabetologia 20, 87–93. 10.1007/BF00262007 7193616

[B9] Berndt S. I. Gustafsson S. Magi R. Ganna A. Wheeler E. Feitosa M. F.. (2013). Genome-wide meta-analysis identifies 11 new loci for anthropometric traits and provides insights into genetic architecture. Nat. Genet. 45, 501–512. 10.1038/ng.2606 23563607 PMC3973018

[B10] Billings L. K. Florez J. C. (2010). The genetics of type 2 diabetes: what have we learned from GWAS? Ann. N.Y. Acad. Sci. 1212, 59–77. 10.1111/j.1749-6632.2010.05838.x 21091714 PMC3057517

[B11] Bluher M. Lubben G. Paschke R. (2003). Analysis of the relationship between the Pro12Ala variant in the PPAR-gamma2 gene and the response rate to therapy with pioglitazone in patients with type 2 diabetes. Diabetes Care 26, 825–831. 10.2337/diacare.26.3.825 12610044

[B12] Bonnefond A. Philippe J. Durand E. Dechaume A. Huyvaert M. Montagne L.. (2012). Whole-exome sequencing and high throughput genotyping identified KCNJ11 as the thirteenth MODY gene. PLoS ONE 7:e37423. 10.1371/journal.pone.0037423 22701567 PMC3372463

[B13] Breier B. H. Vickers M. H. Ikenasio B. A. Chan K. Y. Wong W. P. S. (2001). Fetal programming of appetite and obesity. Mol. Cell. Endocrinol. 185, 73–79. 10.1016/S0303-7207(01)00634-7 11738796

[B14] Buil A. Brown A. A. Lappalainen T. Viñuela A. Davies M. N. Zheng H.-F.. (2015). Gene-gene and gene-environment interactions detected by transcriptome sequence analysis in twins. Nat. Genet. 47, 88–91. 10.1038/ng.3162 25436857 PMC4643454

[B15] Burdge G. C. Hanson M. A. Slater-Jefferies J. L. Lillycrop K. A. (2007). Epigenetic regulation of transcription: a mechanism for inducing variations in phenotype (fetal programming) by differences in nutrition during early life? Br. J. Nutr. 97, 1036–1046. 10.1017/S0007114507682920 17381976 PMC2211525

[B16] Carlson C. S. Eberle M. A. Kruglyak L. Nickerson D. A. (2004). Mapping complex disease loci in whole-genome association studies. Nature 429, 446–452. 10.1038/nature02623 15164069

[B17] Cauchi S. El Achhab Y. Choquet H. Dina C. Krempler F. Weitgasser R.. (2007). TCF7L2 is reproducibly associated with type 2 diabetes in various ethnic groups: a global meta-analysis. J. Mol. Med. 85, 777–782. 10.1007/s00109-007-0203-4 17476472

[B18] Chauhan G. Spurgeon C. J. Tabassum R. Bhaskar S. Kulkarni S. R. Mahajan A.. (2010). Impact of common variants of PPARG, KCNJ11, TCF7L2, SLC30A8, HHEX, CDKN2A, IGF2BP2, and CDKAL1 on the risk of type 2 diabetes in 5,164 Indians. Diabetes 59, 2068–2074. 10.2337/db09-1386 20424228 PMC2911051

[B19] Chen P. Ong R. T.-H. Tay W.-T. Sim X. Ali M. Xu H.. (2013). A study assessing the association of glycated hemoglobin a1C (HbA1C) associated variants with HbA1C, chronic kidney disease and diabetic retinopathy in populations of asian ancestry. PLoS ONE 8:e79767. 10.1371/journal.pone.0079767 24244560 PMC3820602

[B20] Cho Y. S. Chen C.-H. Hu C. Long J. Ong R. T. H. Sim X.. (2012). Meta-analysis of genome-wide association studies identifies eight new loci for type 2 diabetes in east Asians. Nat. Genet. 44, 67–72. 10.1038/ng.1019 22158537 PMC3582398

[B21] Cirulli E. T. Goldstein D. B. (2010). Uncovering the roles of rare variants in common disease through whole-genome sequencing. Nat. Rev. Genet. 11, 415–425. 10.1038/nrg2779 20479773

[B22] Claussnitzer M. Dankel S. N. Klocke B. Grallert H. Glunk V. Berulava T.. (2014). Leveraging cross-species transcription factor binding site patterns: from diabetes risk loci to disease mechanisms. Cell 156, 343–358. 10.1016/j.cell.2013.10.058 24439387 PMC7116609

[B23] Cooper D. N. Krawczak M. Polychronakos C. Tyler-Smith C. Kehrer-Sawatzki H. (2013). Where genotype is not predictive of phenotype: towards an understanding of the molecular basis of reduced penetrance in human inherited disease. Hum. Genet. 132, 1077–1130. 10.1007/s00439-013-1331-2 23820649 PMC3778950

[B24] Dayeh T. A. Olsson A. H. Volkov P. Almgren P. Ronn T. Ling C. (2013). Identification of CpG-SNPs associated with type 2 diabetes and differential DNA methylation in human pancreatic islets. Diabetologia 56, 1036–1046. 10.1007/s00125-012-2815-7 23462794 PMC3622750

[B25] Diabetes Control Complications Trial. (2005). Intensive diabetes treatment and cardiovascular disease in patients with type 1 diabetes. N. Engl. J. Med. 353, 2643. 10.1056/NEJMoa052187 16371630 PMC2637991

[B26] Dina C. Meyre D. Gallina S. Durand E. Körner A. Jacobson P.. (2007). Variation in FTO contributes to childhood obesity and severe adult obesity. Nat. Genet. 39, 724–726. 10.1038/ng2048 17496892

[B27] Dipple K. M. McCabe E. R. B. (2000a). Modifier genes convert “simple” Mendelian disorders to complex traits. Mol. Genet. Metab. 71, 43–50. 10.1006/mgme.2000.3052 11001794

[B28] Dipple K. M. McCabe E. R. B. (2000b). Phenotypes of patients with “simple” Mendelian disorders are complex traits: thresholds, modifiers, and systems dynamics. Am. J. Hum. Genet. 66, 1729–1735. 10.1086/302938 10793008 PMC1378056

[B29] Dupuis J. E. Langenberg C. Prokopenko I. Saxena R. Soranzo N. Jackson A. U.. (2010). New genetic loci implicated in fasting glucose homeostasis and their impact on type 2 diabetes risk. Nat. Genet. 42, 105–116. 10.1038/ng.520 20081858 PMC3018764

[B30] Edghill E. L. Bingham C. Ellard S. Hattersley A. T. (2006). Mutations in hepatocyte nuclear factor-1β and their related phenotypes. J. Med. Genet. 43, 84–90. 10.1136/jmg.2005.032854 15930087 PMC2564507

[B31] Edghill E. L. Flanagan S. E. Ellard S. (2010). Permanent neonatal diabetes due to activating mutations in ABCC8 and KCNJ11. Rev. Endocr. Metab. Disord. 11, 193–198. 10.1007/s11154-010-9149-x 20922570

[B32] Ellard S. Allen H. L. De Franco E. Flanagan S. E. Hysenaj G. Colclough K.. (2013). Improved genetic testing for monogenic diabetes using targeted next-generation sequencing. Diabetologia 56, 1958–1963. 10.1007/s00125-013-2962-5 23771172 PMC3737433

[B33] Elliott H. R. Walia G. K. Duggirala A. Groom A. Reddy S. U. Chandak G. R. (2013). Migration and DNA methylation: a comparison of methylation patterns in type 2 diabetes susceptibility genes between indians and europeans. J. Diab. Res. Clin. Metab. 2, 6 10.7243/2050-0866-2-6 PMC4835020 27099715

[B34] Fajans S. S. Brown M. B. (1993). Administration of sulfonylureas can increase glucose-induced insulin secretion for decades in patients with maturity-onset diabetes of the young. Diabetes Care 16, 1254–1261. 10.2337/diacare.16.9.1254 8404429

[B35] Flanagan S. E. Patch A.-M. Mackay D. J. G. Edghill E. L. Gloyn A. L. Robinson D.. (2007). Mutations in ATP-sensitive K+ channel genes cause transient neonatal diabetes and permanent diabetes in childhood or adulthood. Diabetes 56, 1930–1937. 10.2337/db07-0043 17446535 PMC7611811

[B36] Flannick J. Thorleifsson G. Beer N. L. Jacobs S. B. R. Grarup N. Burtt N. P.. (2014). Loss-of-function mutations in SLC30A8 protect against type 2 diabetes. Nat. Genet. 46, 357–363. 10.1038/ng.2915 24584071 PMC4051628

[B37] Florez J. C. Hirschhorn J. Altshuler D. (2003). The inherited basis of diabetes mellitus: implications for the genetic analysis of complex traits. Annu. Rev. Genom. Hum. G 4, 257–291. 10.1146/annurev.genom.4.070802.110436 14527304

[B38] Florez J. C. Jablonski K. A. McAteer J. B. Franks P. W. Mason C. C. Mather K. (2012). Effects of genetic variants previously associated with fasting glucose and insulin in the Diabetes Prevention Program. PLoS ONE 7:e44424 10.1371/journal.pone.0044424 22984506 PMC3439414

[B39] Fogarty M. P. Cannon M. E. Vadlamudi S. Gaulton K. J. Mohlke K. L. (2014). Identification of a regulatory variant that binds FOXA1 and FOXA2 at the CDC123/CAMK1D type 2 diabetes GWAS locus. PLoS Genet. 10:e1004633. 10.1371/journal.pgen.1004633 25211022 PMC4161327

[B40] Fogarty M. P. Panhuis T. M. Vadlamudi S. Buchkovich M. L. Mohlke K. L. (2013). Allele-Specific Transcriptional activity at type 2 diabetes-associated single nucleotide polymorphisms in regions of pancreatic islet open chromatin at the JAZF1 Locus. Diabetes 62, 1756–1762. 10.2337/db12-0972 23328127 PMC3636602

[B41] Fonseca S. G. Fukuma M. Lipson K. L. Nguyen L. X. Allen J. R. Oka Y.. (2005). WFS1 is a novel component of the unfolded protein response and maintains homeostasis of the endoplasmic reticulum in pancreatic β-cells. J. Biol. Chem. 280, 39609–39615. 10.1074/jbc.M507426200 16195229

[B42] Forbes J. M. Cooper M. E. (2013). Mechanisms of diabetic complications. Physiol. Rev. 93, 137–188. 10.1152/physrev.00045.2011 23303908

[B43] Frayling T. M. Timpson N. J. Weedon M. N. Zeggini E. Freathy R. M. Lindgren C. M.. (2007). A common variant in the FTO gene is associated with body mass index and predisposes to childhood and adult obesity. Science 316, 889–894. 10.1126/science.1141634 17434869 PMC2646098

[B44] Gallou-Kabani C. Gabory A. Tost J. Karimi M. Mayeur S. Lesage J.. (2010). Sex-and diet-specific changes of imprinted gene expression and DNA methylation in mouse placenta under a high-fat diet. PLoS ONE 5:e14398. 10.1371/journal.pone.0014398 21200436 PMC3006175

[B45] Gloyn A. L. Diatloff-Zito C. Edghill E. L. Bellanne-Chantelot C. Nivot S. Coutant R.. (2006). KCNJ11 activating mutations are associated with developmental delay, epilepsy and neonatal diabetes syndrome and other neurological features. Eur. J. Hum. Genet. 14, 824–830. 10.1038/sj.ejhg.5201629 16670688

[B46] Gloyn A. L. Hashim Y. Ashcroft S. J. H. Ashfield R. Wiltshire S. Turner R. C. (2001). Association studies of variants in promoter and coding regions of beta-cell ATP-sensitive K-channel genes SUR1 and Kir6. 2 with Type 2 diabetes mellitus (UKPDS 53). Diabet. Med. 18, 206–212. 10.1046/j.1464-5491.2001.00449.x 11318841

[B47] Gloyn A. L. Pearson E. R. Antcliff J. F. Proks P. Bruining G. J. Slingerland A. S.. (2004). Activating mutations in the gene encoding the ATP-sensitive potassium-channel subunit Kir6. 2 and permanent neonatal diabetes. N. Engl. J. Med. 350, 1838–1849. 10.1056/NEJMoa032922 15115830

[B48] Gong Z. C. Huang Q. Dai X. P. Lei G. H. Lu H. B. Yin J. Y.. (2012). NeuroD1 A45T and PAX4 R121W polymorphisms are associated with plasma glucose level of repaglinide monotherapy in Chinese patients with type 2 diabetes. Br. J. Clin. Pharmacol. 74, 501–509. 10.1111/j.1365-2125.2012.04202.x 22296034 PMC3477351

[B49] Gonzalez-Bulnes A. Ovilo C. (2011). Genetic basis, nutritional challenges and adaptive responses in the prenatal origin of obesity and type-2 diabetes. Curr. Diabetes Rev. 8, 144–154. 10.2174/157339912799424537 22283677

[B50] Greeley S. A. W. Naylor R. N. Philipson L. H. Bell G. I. (2011). Neonatal diabetes: an expanding list of genes allows for improved diagnosis and treatment. Curr. Diab. Rep. 11, 519–532. 10.1007/s11892-011-0234-7 21993633 PMC3226065

[B51] Group UKPDS. (1998). Intensive blood-glucose control with sulphonylureas or insulin compared with conventional treatment and risk of complications in patients with type 2 diabetes (UKPDS 33). Lancet 352, 837–853. 10.1016/S0140-6736(98)07019-6 9742976

[B52] Han X. Luo Y. Ren Q. Zhang X. Wang F. Sun X.. (2010). Implication of genetic variants near SLC30A8, HHEX, CDKAL1, CDKN2A/B, IGF2BP2, FTO, TCF2, KCNQ1, and WFS1 in type 2 diabetes in a Chinese population. BMC Med. Genet. 11, 81–90. 10.1186/1471-2350-11-81 20509872 PMC2896346

[B53] He Y. Y. Zhang R. Shao X. Y. Hu C. Wang C. R. Lu J. X.. (2008). Association of KCNJ11 and ABCC8 genetic polymorphisms with response to repaglinide in Chinese diabetic patients. Acta Pharmacol. Sin. 29, 983–989. 10.1111/j.1745-7254.2008.00840.x 18664331

[B54] Hegele R. A. (2003). Monogenic forms of insulin resistance: apertures that expose the common metabolic syndrome. Trends Endocrinol. Metab. 14, 371–377. 10.1016/S1043-2760(03)00142-5 14516935

[B55] Hu C. Zhang R. Wang C. Wang J. Ma X. Lu J.. (2009). PPARG, KCNJ11, CDKAL1, CDKN2A-CDKN2B, IDE-KIF11-HHEX, IGF2BP2 and SLC30A8 are associated with type 2 diabetes in a chinese population. PLoS ONE 4:e7643. 10.1371/journal.pone.0007643 19862325 PMC2763267

[B56] Ingram V. M. (1957). Gene mutations in human haemoglobin: the chemical difference between normal and sickle cell haemoglobin. Nature 180, 326–328. 10.1038/180326a0 13464827

[B57] Ismail-Beigi F. Craven T. Banerji M. A. Basile J. Calles J. Cohen R. M.. (2011). Effect of intensive treatment of hyperglycaemia on microvascular outcomes in type 2 diabetes: an analysis of the ACCORD randomised trial. Lancet 376, 419–430. 10.1016/S0140-6736(10)60576-4 20594588 PMC4123233

[B58] Jablonski K. A. McAteer J. B. de Bakker P. I. W. Franks P. W. Pollin T. I. Hanson R. L.. (2010). Common variants in 40 genes assessed for diabetes incidence and response to metformin and lifestyle intervention in the diabetes prevention program. Diabetes 59, 2672–2681. 10.2337/db10-0543 20682687 PMC3279522

[B59] Jaenisch R. Bird A. (2003). Epigenetic regulation of gene expression: how the genome integrates intrinsic and environmental signals. Nat. Genet. 33, 245–254. 10.1038/ng1089 12610534

[B60] Johansson S. Irgens H. Chudasama K. K. Molnes J. Aerts J. Roque F. S.. (2012). Exome sequencing and genetic testing for MODY. PLoS ONE 7:e38050. 10.1371/journal.pone.0037423 22662265 PMC3360646

[B61] Knip M. Veijola R. Virtanen S. M. Hyöty H. Vaarala O. Akerblom H. K. (2005). Environmental triggers and determinants of type 1 diabetes. Diabetes 54, S125–S136. 10.2337/diabetes.54.suppl_2.S125 16306330

[B62] Kobberling J. Tillil H. (1982). Empirical risk figures for first degree relatives of non-insulin dependent diabetics, in The Genetics of Diabetes Mellitus, eds Kobberling J. Tattersall R. (London: Academic Press), 201–09.

[B63] Kooner J. S. Saleheen D. Sim X. Sehmi J. Zhang W. Frossard P.. (2011). Genome-wide association study in individuals of South Asian ancestry identifies six new type 2 diabetes susceptibility loci. Nat. Genet. 43, 984–989. 10.1038/ng.921 21874001 PMC3773920

[B64] Ku C.-S. Naidoo N. Pawitan Y. (2011). Revisiting Mendelian disorders through exome sequencing. Hum. Genet. 129, 351–370. 10.1007/s00439-011-0964-2 21331778

[B65] Kulkarni A. Dangat K. Kale A. Sable P. Chavan-Gautam P. Joshi S. (2011). Effects of altered maternal folic acid, vitamin B12 and docosahexaenoic acid on placental global DNA methylation patterns in Wistar rats. PLoS ONE 6:e17706. 10.1371/journal.pone.0017706 21423696 PMC3053375

[B66] Laukkanen O. Pihlajamaki J. Lindstrom J. Eriksson J. Valle T. T. Hamalainen H.. (2004). Polymorphisms of the SUR1 (ABCC8) and Kir6. 2 (KCNJ11) genes predict the conversion from impaired glucose tolerance to type 2 diabetes. The Finnish diabetes prevention study. J. Clin. Endocr. Metab. 89, 6286–6290. 10.1210/jc.2004-1204 15579791

[B67] Lee Y.-H. Kang E. S. Kim S. H. Han S. J. Kim C. H. Kim H. J.. (2008). Association between polymorphisms in SLC30A8, HHEX, CDKN2A/B, IGF2BP2, FTO, WFS1, CDKAL1, KCNQ1 and type 2 diabetes in the Korean population. J. Hum. Genet. 53, 991–998. 10.1007/s10038-008-0341-8 18991055

[B68] Lettre G. Sankaran V. G. Bezerra M. A. C. Araujo A. S. Uda M. Sanna S.. (2008). DNA polymorphisms at the BCL11A, HBS1L-MYB, and β-globin loci associate with fetal hemoglobin levels and pain crises in sickle cell disease. Proc. Natl. Acad. Sci. U.S.A. 105, 11869–11874. 10.1073/pnas.0804799105 18667698 PMC2491485

[B69] Ling C. Groop L. (2009). Epigenetics: a molecular link between environmental factors and type 2 diabetes. Diabetes 58, 2718–2725. 10.2337/db09-1003 19940235 PMC2780862

[B70] Locke A. E. Kahali B. Berndt S. I. Justice A. E. Pers T. H. Day F. R.. (2015). Genetic studies of body mass index yield new insights for obesity biology. Nature 518, 197–206. 10.1038/nature14177 25673413 PMC4382211

[B71] Ma R. C. W. Hu C. Tam C. H. Zhang R. Kwan P. Leung T. F.. (2013). Genome-wide association study in a Chinese population identifies a susceptibility locus for type 2 diabetes at 7q32 near PAX4. Diabetologia 56, 1291–1305. 10.1007/s00125-013-2874-4 23532257 PMC3648687

[B72] Mackay D. J. G. Callaway J. L. A. Marks S. M. White H. E. Acerini C. L. Boonen S. E.. (2008). Hypomethylation of multiple imprinted loci in individuals with transient neonatal diabetes is associated with mutations in ZFP57. Nat. Genet. 40, 949–951. 10.1038/ng.187 18622393

[B73] Majithia A. R. Flannick J. Shahinian P. Guo M. Bray M.-A. Fontanillas P.. (2014). Rare variants in PPARG with decreased activity in adipocyte differentiation are associated with increased risk of type 2 diabetes. Proc. Natl. Acad. Sci. U.S.A. 111, 13127–13132. 10.1073/pnas.1410428111 25157153 PMC4246964

[B74] Manning A. K. Hivert M.-F. Scott R. A. Grimsby J. L. Bouatia-Naji N. Chen H.. (2012). A genome-wide approach accounting for body mass index identifies genetic variants influencing fasting glycemic traits and insulin resistance. Nat. Genet. 44, 659–669. 10.1038/ng.2274 22581228 PMC3613127

[B75] Manolio T. A. Collins F. S. Cox N. J. Goldstein D. B. Hindorff L. A. Hunter D. J.. (2009). Finding the missing heritability of complex diseases. Nature 461, 747–753. 10.1038/nature08494 19812666 PMC2831613

[B76] Mathers C. D. Loncar D. (2006). Projections of global mortality and burden of disease from 2002 to 2030. PLoS Med. 3:e442. 10.1371/journal.pmed.0030442 17132052 PMC1664601

[B77] Matschinsky F. M. (1990). Glucokinase as glucose sensor and metabolic signal generator in pancreatic β-cells and hepatocytes. Diabetes 36, 647–652. 10.2337/diab.39.6.647 2189759

[B78] Matschinsky F. M. (2002). Regulation of pancreatic β-cell glucokinase from basics to therapeutics. Diabetes 51, S394–S404. 10.2337/diabetes.51.2007.S394 12475782

[B79] McCarthy M. I. (2004). Progress in defining the molecular basis of type 2 diabetes mellitus through susceptibility-gene identification. Hum. Mol. Genet. 13, R33–R41. 10.1093/hmg/ddh057 14722160

[B80] Menzel S. Thein S. L. (2009). Genetic architecture of hemoglobin F control. Curr. Opin. Hematol. 16, 179–186. 10.1097/MOH.0b013e328329d07a 19475730

[B81] Mlynarski W. Tarasov A. I. Gach A. Girard C. A. Pietrzak I. Zubcevic L.. (2007). Sulfonylurea improves CNS function in a case of intermediate DEND syndrome caused by a mutation in KCNJ11. Nat. Clin. Pract. Neurol. 3, 640–645. 10.1038/ncpneuro0640 17982434

[B82] Morris A. P. Voight B. F. Teslovich T. M. Ferreira T. Segre A. V. Steinthorsdottir V.. (2012). Large-scale association analysis provides insights into the genetic architecture and pathophysiology of type 2 diabetes. Nat. Genet. 44, 981. 10.1038/ng.2383 22885922 PMC3442244

[B83] Muller Y. L. Piaggi P. Hoffman D. Huang K. Gene B. Kobes S.. (2014). Common genetic variation in the glucokinase gene (GCK) is associated with type 2 diabetes and rates of carbohydrate oxidation and energy expenditure. Diabetologia 57, 1382–1390. 10.1007/s00125-014-3234-8 24728127 PMC4052004

[B84] Murphy R. Ellard S. Hattersley A. T. (2008). Clinical implications of a molecular genetic classification of monogenic beta-cell diabetes. Nat. Clin. Pract. Endocrinol. Metab. 4, 200–213. 10.1038/ncpendmet0778 18301398

[B85] Naylor R. Philipson L. H. (2011). Who should have genetic testing for maturity-onset diabetes of the young? Clin. Endocrinol. (Oxf). 75, 422–426. 10.1111/j.1365-2265.2011.04049.x 21521318

[B86] Ng M. C. Park K. S. Oh B. Tam C. H. Cho Y. M. Shin H. D.. (2008). Implication of genetic variants near TCF7L2, SLC30A8, HHEX, CDKAL1, CDKN2A/B, IGF2BP2, and FTO in type 2 diabetes and obesity in 6,719 Asians. Diabetes 57, 2226–2233. 10.2337/db07-1583 18469204 PMC2494677

[B87] Ng S.-F. Lin R. C. Y. Laybutt D. R. Barres R. Owens J. A. Morris M. J. (2010). Chronic high-fat diet in fathers programs β-cell dysfunction in female rat offspring. Nature 467, 963–966. 10.1038/nature09491 20962845

[B88] Nicolino M. Claiborn K. C. Senee V. Boland A. Stoffers D. A. Julier C. (2010). A novel hypomorphic PDX1 mutation responsible for permanent neonatal diabetes with subclinical exocrine deficiency. Diabetes 59, 733–740. 10.2337/db09-1284 20009086 PMC2828654

[B89] Nilsson E. Jansson P. A. Perfilyev A. Volkov P. Pedersen M. Svensson M. K.. (2014). Altered DNA methylation and differential expression of genes influencing metabolism and inflammation in adipose tissue from subjects with type 2 diabetes. Diabetes 63, 2962–2976. 10.2337/db13-1459 24812430

[B90] Oliver-Krasinski J. M. Kasner M. T. Yang J. Crutchlow M. F. Rustgi A. K. Kaestner K. H.. (2009). The diabetes gene Pdx1 regulates the transcriptional network of pancreatic endocrine progenitor cells in mice. J. Clin. Invest. 119, 1888. 10.1172/JCI37028 19487809 PMC2701861

[B91] Onengut-Gumuscu S. Chen W.-M. Burren O. Cooper N. J. Quinlan A. R. Mychaleckyj J. C.. (2015). Fine mapping of type 1 diabetes susceptibility loci and evidence for colocalization of causal variants with lymphoid gene enhancers. Nat. Genet. 47, 381–386. 10.1038/ng.3245 25751624 PMC4380767

[B92] Owen K. R. (2013). Monogenic diabetes: old and new approaches to diagnosis. Clin. Med. (Northfield. Il). 13, 278–281. 10.7861/clinmedicine.13-3-278 23760703 PMC5922673

[B93] Ozanne S. E. Hales C. N. (2002). Early programming of glucose-insulin metabolism. Trends Endocrinol. Metab. 13, 368–373. 10.1016/S1043-2760(02)00666-5 12367817

[B94] Patch A. M. Flanagan S. E. Boustred C. Hattersley A. T. Ellard S. (2007). Mutations in the ABCC8 gene encoding the SUR1 subunit of the KATP channel cause transient neonatal diabetes, permanent neonatal diabetes or permanent diabetes diagnosed outside the neonatal period. Diabetes Obes. Metab. 9, 28–39. 10.1111/j.1463-1326.2007.00772.x 17919176 PMC7611803

[B95] Pearson E. R. Boj S. F. Steele A. M. Barrett T. Stals K. Shield J. P.. (2007). Macrosomia and hyperinsulinaemic hypoglycaemia in patients with heterozygous mutations in the HNF4A gene. PLoS Med. 4:e118. 10.1371/journal.pmed.0040118 17407387 PMC1845156

[B96] Pearson E. R. Flechtner I. NjoIstad P. R. Malecki M. T. Flanagan S. E. Larkin B.. (2006). Switching from insulin to oral sulfonylureas in patients with diabetes due to Kir6. 2 mutations. N. Engl. J. Med. 355, 467–477. 10.1056/NEJMoa061759 16885550

[B97] Pearson E. R. Liddell W. G. Shepherd M. Corrall R. J. Hattersley A. T. (2000). Sensitivity to sulphonylureas in patients with hepatocyte nuclear factor-1alpha gene mutations: evidence for pharmacogenetics in diabetes. Diabet. Med. 17, 543 45. 10.1046/j.1464-5491.2000.00305.x 10972586

[B98] Pihoker C. Gilliam L. K. Ellard S. Dabelea D. Davis C. Dolan L. M.. (2013). Prevalence, characteristics and clinical diagnosis of maturity onset diabetes of the young due to mutations in HNF1A, HNF4A, and glucokinase: results from the SEARCH for Diabetes in Youth. J. Clin. Endocr. Metab. 98, 4055–4062. 10.1210/jc.2013-1279 23771925 PMC3790621

[B99] Pritchard J. K. Cox N. J. (2002). The allelic architecture of human disease genes: common disease-common variant or not? Hum. Mol. Genet. 11, 2417–2423. 10.1093/hmg/11.20.2417 12351577

[B100] Rafiq M. Flanagan S. E. Patch A.-M. Shields B. M. Ellard S. Hattersley A. T. (2008). Effective treatment with oral sulfonylureas in patients with diabetes due to sulfonylurea receptor 1 (SUR1) mutations. Diabetes Care 31, 204–209. 10.2337/dc07-1785 18025408 PMC7611807

[B101] Randall J. C. Winkler T. W. Kutalik Z. Berndt S. I. Jackson A. U. Monda K. L.. (2013). Sex-stratified genome-wide association studies including 270,000 individuals show sexual dimorphism in genetic loci for anthropometric traits. PLoS Genet. 9:e1003500. 10.1371/journal.pgen.1003500 23754948 PMC3674993

[B102] Rees S. D. Hydrie M. Z. I. O'Hare J. P. Kumar S. Shera A. S. Basit A.. (2011). Effects of 16 genetic variants on fasting glucose and type 2 diabetes in South Asians: ADCY5 and GLIS3 variants may predispose to type 2 diabetes. PLoS ONE 6:e24710. 10.1371/journal.pone.0024710 21949744 PMC3176767

[B103] Reich D. E. Lander E. S. (2001). On the allelic spectrum of human disease. Trends Genet. 17, 502–510. 10.1016/S0168-9525(01)02410-6 11525833

[B104] Rong R. Hanson R. L. Ortiz D. Wiedrich C. Kobes S. Knowler W. C.. (2009). Association analysis of variation in/near FTO, CDKAL1, SLC30A8, HHEX, EXT2, IGF2BP2, LOC387761 and CDKN2B with type 2 diabetes and related quantitative traits in Pima Indians. Diabetes 58, 478–488. 10.2337/db08-0877 19008344 PMC2628623

[B105] Sagen J. V. Raeder H. Hathout E. Shehadeh N. Gudmundsson K. Baevre H.. (2004). Permanent Neonatal Diabetes due to Mutations in KCNJ11 Encoding Kir6. 2 Patient Characteristics and Initial Response to Sulfonylurea Therapy. Diabetes 53, 2713–2718. 10.2337/diabetes.53.10.2713 15448106

[B106] Sandhu M. S. Weedon M. N. Fawcett K. A. Wasson J. Debenham S. L. Daly A.. (2007). Common variants in WFS1 confer risk of type 2 diabetes. Nat. Genet. 39, 951–953. 10.1038/ng2067 17603484 PMC2672152

[B107] Sandovici I. Smith N. H. Nitert M. D. Ackers-Johnson M. Uribe-Lewis S. Ito Y.. (2011). Maternal diet and aging alter the epigenetic control of a promoter-enhancer interaction at the *Hnf4a* gene in rat pancreatic islets. Proc. Natl. Acad. Sci. U.S.A. 108, 5449–5454. 10.1073/pnas.1019007108 21385945 PMC3069181

[B108] Saxena R. Voight B. F. Lyssenko V. Burtt N. P. de Bakker P. I. W. Chen H.. (2007). Genome-wide association analysis identifies loci for type 2 diabetes and triglyceride levels. Science 316, 1331–1336. 10.1126/science.1142358 17463246

[B109] Schwitzgebel V. M. (2014). Many faces of monogenic diabetes. J. Diabetes Investig. 5, 121–133. 10.1111/jdi.12197 24843749 PMC4023572

[B110] Scott R. A. Lagou V. Welch R. P. Wheeler E. Montasser M. E. Luan J. A.. (2012). Large-scale association analyses identify new loci influencing glycemic traits and provide insight into the underlying biological pathways. Nat. Genet. 44, 991–1005. 10.1038/ng.2385 22885924 PMC3433394

[B111] Scuteri A. Sanna S. Chen W.-M. Uda M. Albai G. Strait J.. (2007). Genome-wide association scan shows genetic variants in the FTO gene are associated with obesity-related traits. PLoS Genet. 3:e115. 10.1371/journal.pgen.0030115 17658951 PMC1934391

[B112] Sedgewick A. E. Timofeev N. Sebastiani P. So J. C. C. Ma E. S. K. Chan L. C.. (2008). BCL11A is a major HbF quantitative trait locus in three different populations with beta-hemoglobinopathies. Blood Cells Mol. Dis. 41, 255–258. 10.1016/j.bcmd.2008.06.007 18691915 PMC4100606

[B113] Servitja J. M. Pignatelli M. Maestro M. A. Cardalda C. Boj S. F. Lozano J.. (2009). Hnf1alpha (MODY3) controls tissue-specific transcriptional programs and exerts opposed effects on cell growth in pancreatic islets and liver. Mol. Cell. Biol. 29, 2945–2959. 10.1128/MCB.01389-08 19289501 PMC2682018

[B114] Shields B. M. Hicks S. Shepherd M. H. Colclough K. Hattersley A. T. Ellard S. (2010). Maturity-onset diabetes of the young (MODY): how many cases are we missing? Diabetologia 53, 2504–2508. 10.1007/s00125-010-1799-4 20499044

[B115] Shungin D. Winkler T. W. Croteau-Chonka D. C. Ferreira T. Locke A. E. Magi R.. (2015). New genetic loci link adipose and insulin biology to body fat distribution. Nature 518, 187–196. 10.1038/nature14132 25673412 PMC4338562

[B116] Sidransky E. (2006). Heterozygosity for a Mendelian disorder as a risk factor for complex disease. Clin. Genet. 70, 275–282. 10.1111/j.1399-0004.2006.00688.x 16965318

[B117] Sladek R. Rocheleau G. Rung J. Dina C. Shen L. Serre D.. (2007). A genome-wide association study identifies novel risk loci for type 2 diabetes. Nature 445, 881–885. 10.1038/nature05616 17293876

[B118] Snoeck A. Remacle C. Reusens B. Hoet J. J. (1990). Effect of a low protein diet during pregnancy on the fetal rat endocrine pancreas. Biol. Neonate 57, 107–118. 10.1159/000243170 2178691

[B119] Soranzo N. Sanna S. Wheeler E. Gieger C. Radke D. Dupuis J.. (2010). Common variants at 10 genomic loci influence hemoglobin A1C levels via glycemic and nonglycemic pathways. Diabetes 59, 3229–3239. 10.2337/db10-0502 20858683 PMC2992787

[B120] Steele A. M. Shields B. M. Wensley K. J. Colclough K. Ellard S. Hattersley A. T. (2014). Prevalence of vascular complications among patients with glucokinase mutations and prolonged, mild hyperglycemia. JAMA 311, 279–286. 10.1001/jama.2013.283980 24430320

[B121] Steinberg M. H. (2005). Predicting clinical severity in sickle cell anaemia. Br. J. Haematol. 129, 465–481. 10.1111/j.1365-2141.2005.05411.x 15877729

[B122] Steinthorsdottir V. Thorleifsson G. Sulem P. Helgason H. Grarup N. Sigurdsson A.. (2014). Identification of low-frequency and rare sequence variants associated with elevated or reduced risk of type 2 diabetes. Nat. Genet. 46, 294–298. 10.1038/ng.2882 24464100

[B123] Stoy J. Edghill E. L. Flanagan S. E. Ye H. Paz V. P. Pluzhnikov A.. (2007). Insulin gene mutations as a cause of permanent neonatal diabetes. Proc. Natl. Acad. Sci.U.S.A. 104, 15040–15044. 10.1073/pnas.0707291104 17855560 PMC1986609

[B124] Strawbridge R. J. Dupuis J. Prokopenko I. Barker A. Ahlqvist E. Rybin D.. (2010). Genome-wide association identifies nine common variants associated with fasting proinsulin levels and provides new insights into the pathophysiology of type 2 diabetes. Diabetes 60, 2624–2634. 10.2337/db11-0415 21873549 PMC3178302

[B125] Stride A. Vaxillaire M. Tuomi T. Barbetti F. Njolstad P. R. Hansen T.. (2002). The genetic abnormality in the beta cell determines the response to an oral glucose load. Diabetologia 45, 427–435. 10.1007/s00125-001-0770-9 11914749

[B126] Takei D. Ishihara H. Yamaguchi S. Yamada T. Tamura A. Katagiri H.. (2006). WFS1 protein modulates the free Ca 2+ concentration in the endoplasmic reticulum. FEBS Lett. 580, 5635–5640. 10.1016/j.febslet.2006.09.007 16989814

[B127] Temple I. K. Shield J. P. H. (2002). Transient neonatal diabetes, a disorder of imprinting. J. Med. Genet. 39, 872–875. 10.1136/jmg.39.12.872 12471198 PMC1757233

[B128] Thein S. L. Menzel S. Lathrop M. Garner C. (2009). Control of fetal hemoglobin: new insights emerging from genomics and clinical implications. Hum. Mol. Genet. 18, R216–R223. 10.1093/hmg/ddp401 19808799 PMC2758709

[B129] Thomas C. C. Philipson L. H. (2015). Update on diabetes classification. Med. Clin. North Am. 99, 1–16. 10.1016/j.mcna.2014.08.015 25456640

[B130] Uda M. Galanello R. Sanna S. Lettre G. Sankaran V. G. Chen W.. (2008). Genome-wide association study shows BCL11A associated with persistent fetal hemoglobin and amelioration of the phenotype of β-thalassemia. Proc. Natl. Acad. Sci.U.S.A. 105, 1620–1625. 10.1073/pnas.0711566105 18245381 PMC2234194

[B131] Vaarala O. Atkinson M. A. Neu J. (2008). The “perfect storm” for type 1 diabetes: the complex interplay between intestinal microbiota, gut permeability, and mucosal immunity. Diabetes 57, 2555–2562. 10.2337/db08-0331 18820210 PMC2551660

[B132] van der Zwaag A. M. Weinreich S. S. Bosma A. R. Rigter T. Losekoot M. Henneman L.. (2015). Current and best practices of genetic testing for maturity onset diabetes of the young: views of professional experts. Public Health Genomics 18, 52–59. 10.1159/000367963 25341961

[B133] Voight B. F. Scott L. J. Steinthorsdottir V. Morris A. P. Dina C. Welch R. P.. (2010). Twelve type 2 diabetes susceptibility loci identified through large-scale association analysis. Nat. Genet. 42, 579–589. 10.1038/ng.609 20581827 PMC3080658

[B134] Waki H. Yamauchi T. Kadowaki T. (2012). The epigenome and its role in diabetes. Curr. Diab. Rep. 12, 673–685. 10.1007/s11892-012-0328-x 23070559

[B135] Weatherall D. J. (2000). Single gene disorders or complex traits: lessons from the thalassaemias and other monogenic diseases. BMJ 321, 1117. 10.1136/bmj.321.7269.1117 11061733 PMC1118897

[B136] Wegner L. Andersen G. Sparso T. Grarup N. Glumer C. Borch-Johnsen K.. (2007). Common variation in LMNA increases susceptibility to type 2 diabetes and associates with elevated fasting glycemia and estimates of body fat and height in the general population studies of 7,495 danish whites. Diabetes 56, 694–698. 10.2337/db06-0927 17327437

[B137] Wild S. Roglic G. Green A. Sicree R. King H. (2004). Global prevalence of diabetes estimates for the year 2000 and projections for 2030. Diabetes Care 27, 1047–1053. 10.2337/diacare.27.5.1047 15111519

[B138] Winckler W. Weedon M. N. Graham R. R. McCarroll S. A. Purcell S. Almgren P.. (2007). Evaluation of common variants in the six known maturity-onset diabetes of the young (MODY) genes for association with type 2 diabetes. Diabetes 56, 685–693. 10.2337/db06-0202 17327436

[B139] Wu Y. Li H. Loos R. J. Yu Z. Ye X. Chen L.. (2008). Common variants in CDKAL1, CDKN2A/B, IGF2BP2, SLC30A8, and HHEX/IDE genes are associated with type 2 diabetes and impaired fasting glucose in a Chinese Han population. Diabetes 57, 2834–2842. 10.2337/db08-0047 18633108 PMC2551696

[B140] Yajnik C. S. Deshmukh U. S. (2012). Fetal programming: maternal nutrition and role of one-carbon metabolism. Rev. Endocr. Metab. Disord. 13, 121–127. 10.1007/s11154-012-9214-8 22415298

[B141] Yajnik C. S. Deshpande S. S. Jackson A. A. Refsum H. Rao S. Fisher D. J.. (2008). Vitamin B12 and folate concentrations during pregnancy and insulin resistance in the offspring: the Pune Maternal Nutrition Study. Diabetologia 51, 29–38. 10.1007/s00125-007-0793-y 17851649 PMC2100429

[B142] Yajnik C. S. Janipalli C. S. Bhaskar S. Kulkarni S. R. Freathy R. M. Prakash S.. (2009). FTO gene variants are strongly associated with type 2 diabetes in South Asian Indians. Diabetologia 52, 247–252. 10.1007/s00125-008-1186-6 19005641 PMC2658005

[B143] Yang B. T. Dayeh T. A. Kirkpatrick C. L. Taneera J. Kumar R. Groop L.. (2011). Insulin promoter DNA methylation correlates negatively with insulin gene expression and positively with HbA1c levels in human pancreatic islets. Diabetologia 54, 360–367. 10.1007/s00125-010-1967-6 21104225 PMC3017313

[B144] Yang B. T. Dayeh T. A. Volkov P. A. Kirkpatrick C. L. Malmgren S. Jing X.. (2012). Increased DNA methylation and decreased expression of PDX-1 in pancreatic islets from patients with type 2 diabetes. Mol. Endocrinol. 26, 1203–1212. 10.1210/me.2012-1004 22570331 PMC5416998

[B145] Zeggini E. Scott L. J. Saxena R. Voight B. F. Marchini J. L. Hu T.. (2008). Meta-analysis of genome-wide association data and large-scale replication identifies additional susceptibility loci for type 2 diabetes. Nat. Genet. 40, 638–645. 10.1038/ng.120 18372903 PMC2672416

[B146] Zoungas S. De Galan B. E. Ninomiya T. Grobbee D. Hamet P. Heller S.. (2009). Combined effects of routine blood pressure lowering and intensive glucose control on macrovascular and microvascular outcomes in patients with type 2 diabetes new results from the ADVANCE trial. Diabetes Care 32, 2068–2074. 10.2337/dc09-0959 19651921 PMC2768202

