# Supplementary material for: New insights from monogenic diabetes for “common” type 2 diabetes
Source: Front Genet. 2015 Aug 7;6:251. doi: 10.3389/fgene.2015.00251 (PMC4528293; doi:10.3389/fgene.2015.00251)
Supplement: Supplementary file 1 [file Table3.DOC]

**Table 3: Genes/loci associated with type 2 diabetes risk**

| **Gene/loci** | **Gene name** | **Region** | **SNP(s)** | **Nature of variant** | **Ethnicity** | **PubMed ID** |
| --- | --- | --- | --- | --- | --- | --- |
| *ACHE* | Acetylcholinesterase | 7q22.1 | rs7636 | Synonymous | Chinese, Malay, Asian Indian | 21490949 |
| *ADAMTS9* | ADAM metallopeptidase with thrombospondin type 1 motif, 9 | 3p14.1 | rs4607103 | Intron | European | 18372903 |
| *ADCY5* | Adenylate cyclase 5 | 3q21.1 | rs11717195 | Intron | European | 24509480 |
| *ANK1* | Ankyrin 1, erythrocytic | 8p11.21 | rs516946 | Non coding transcript exon | European | 24509480 |
|  |  |  | rs515071 | Splice region | Japanese | 22456796 |
| *AP3S2* | Adaptor-related protein complex 3, sigma 2 subunit | 15q26.1 | rs2028299 | 3 prime UTR | European,East Asian,South Asian | 24509480 |
| *ARAP1* | ArfGAP with RhoGAP domain, ankyrin repeat and PH domain 1; | 11q13.4 | rs1552224 | Intron | European | 24509480 |
| *ARF5, PAX4, SND1* | ADP-ribosylation factor 5; paired box 4; staphylococcal nuclease and tudor domain containing 1 | 7q32.1 | rs10229583 | Downstream gene | Han Chinese, East Asian, Singaporean Malay, Singaporean Indian, European | 23532257 |
| *ARL15* | ADP-ribosylation factor-like 15 | 5q11.2 | rs702634 | Intron | European | 24509480 |
| *BCL11A* | B-cell CLL/lymphoma 11A (zinc finger protein) | 2p16.1 | rs243088, rs243021 | Intergenic,Upstream gene | European | 24509480, 20581827 |
| *C10orf35* | Chromosome 10 open reading frame 35 | 10q22.1 | rs2812533 | Downstream gene | European | 24509480 |
| *C14orf70/LINC00523* | Long intergenic non-protein coding RNA 523 | 14q32.2 | rs730570 | Splice region | Hispanic, European | 21573907 |
| *C2CD4A* | C2 calcium-dependent domain containing 4A | 15q22.2 | rs7163757 | Regulatory region | European | 24509480 |
| *C2CD4A, C2CD4B* | C2 calcium-dependent domain containing 4A ; calcium/calmodulin-dependent protein kinase ID | 15q22.2 | rs7172432 | Intergenic | East Asian | 23945395 |
|  |  |  | rs1436953 | Intergenic | Han Chinese | 21799836 |
|  |  |  | rs7172432 | Intergenic | Japanese, European, East Asian | 23945395 |
| *C2CD4B* | C2 calcium-dependent domain containing 4B | 15q22.2 | rs1436955 | Regulatory region | Chinese, European, East Asian | 20862305 |
| *CENPW* | Centromere protein W | 6q22.32 | rs4273712 | Upstream gene | European | 24509480 |
| *C6orf57* | Chromosome 3 open reading frame, | 6q13 | rs1048886 | Missense | Chinese, Malay, Asian Indian | 21490949 |
| *CDC123* | Cell division cycle 123 | 10p13 | rs11257655 | Regulatory region | European,East Asian, Singaporean Malay, South Asian | 24509480, 22961080 |
| *CDC123,CAMK1D* | Cell division cycle 123; calcium/calmodulin-dependent protein kinase ID | 10p13 | rs10906115 | Intergenic | Chinese, European, East Asian | 20862305 |
|  |  |  | rs12779790 | Intergenic | European | 18372903 |
| *CDKAL1* | CDK5 regulatory subunit associated protein 1-like 1 | 6p22.3 | rs7756992 | Intron | European | 24509480 |
|  |  |  | rs7754840 | Intron | East Asian, Singaporean Malay, South Asian | 23945395, 22961080 |
|  |  |  | rs9295474 | Intron | Chinese, Malay, Asian Indian | 21490949 |
|  |  |  | rs1044083 | Intron | European | 20581827 |
|  |  |  | rs4712523 | Intron | Japanese | 19401414 |
|  |  |  | rs4712524 | Intron | Japanese, East Asian, European | 18711366 |
|  |  |  | rs6931514 | Intron | European | 18372903 |
|  |  |  | rs9465871 | Intron | European | 17554300 |
|  |  |  | rs10946398 | Intron | European | 17463249 |
| *CDKN2A* | Cyclin-dependent kinase inhibitor 2A | 9p21.3 | rs2383208 | Intergenic | Indo-European, Dravidian, European, Japanese,East Asian, Singaporean Malay, South Asian | 23209189 |
| *CDKN2A, CDKN2B* | Cyclin-dependent kinase inhibitor 2A; cyclin-dependent kinase inhibitor 2B (p15, inhibits CDK4) | 9p21.3 | rs10811661 | Intergenic | East Asian,European | 23945395 |
|  |  |  | rs1333051 | Regulatory region | Hispanic, European | 21573907 |
|  |  |  | rs564398 | ncRNA | European | 17463249 |
|  |  |  | rs10965250 | Intergenic | European | 20581827 |
|  |  |  | rs7020996 | Regulatory region | European | 18372903 |
| *CDKN2B* | Cyclin-dependent kinase inhibitor 2B (p15, inhibits CDK4) | 9p21.3 | rs7018475 | Regulatory region | European | 22293688 |
| *CHCHD9* | Coiled-coil-helix-coiled-coil-helix domain containing 2 pseudogene 9 | 9q21.31 | rs13292136 | Intergenic | European | 20581827 |
| *CMIP* | c-Maf inducing protein | 16q23.2 | rs16955379 | Intron | East Asian | 22158537 |
| *CR2* | Complement component (3d/Epstein Barr virus) receptor 2 | 1q32.2 | rs17045328 | Intron | Chinese, Malay, Asian Indian | 21490949 |
| *CRHR2* | Corticotropin releasing hormone receptor 2 | 7p14.3 | rs2284219 | Intron | European | 24509480 |
| *DCD* | Dermcidin | 12q13.2 | rs1153188 | Intergenic | European | 18372903 |
| *DGKB* | Diacylglycerol kinase, beta 90kDa | 7p21.2 | rs17168486 | Intron | European | 24509480 |
| *DNER* | Delta/notch-like EGF repeat containing | 2q36.3 | rs1861612 | Intron | American Indian | 24101674 |
| *DUSP9* | Dual specificity phosphatase 9 | Xq28 | rs5945326 | Intergenic | East Asian,European, Singaporean Malay, South Asian | 23945395, 22961080 |
| *ETV1* | Ets variant 1 | 7p21.2 | rs7795991 | Upstream gene | European | 24509480 |
| *FAF1* | Fas (TNFRSF6) associated factor 1 | 1p32.3 | rs17106184 | Intron | European | 24509480 |
| *FAM58A* | Family with sequence similarity 58, member A | Xq28 | rs12010175 | Intron | East Asian, Singaporean Malay, South Asian | 22961080 |
| *FITM2,R3HDML,HNF4A* | Fat storage-inducing transmembrane protein 2; R3H domain containing-like; hepatocyte nuclear factor 4 alpha | 20q13.12 | rs6017317 | Intergenic | East Asian | 22158537 |
| *FLJ16165/PAPL* | Iron/zinc purple acid phosphatase-like protein | 19q13.2 | rs472265 | Intron | Chinese, Malay, Asian Indian | 21490949 |
| *FTO* | Fat mass and obesity associated | 16q12.2 | rs9936385 | Intron | European | 24509480 |
|  |  |  | rs8050136 | Intron | Indo-European, Dravidian, European | 23209189 |
|  |  |  | rs11642841 | Intron | European | 20581827 |
|  |  |  | rs8050136 | Intron | European | 19056611, 18372900 |
|  |  |  | rs9939609 | Intron | European | 17554300 |
| *GABRA4, COX7B2* | Gamma-aminobutyric acid (GABA) A receptor, alpha 4 ; cytochrome c oxidase subunit VIIb2 | 4p12 | rs2055942 | Intron | Arab | 23937595 |
| *GALNTL4* | UDP-N-acetyl-alpha-D-galactosamine:polypeptide N-acetylgalactosaminyltransferase-like 4 | 11p15.3 | rs2722769 | Intergenic | African American | 22238593 |
| *GCC1,PAX4* | GRIP and coiled-coil domain containing 1; paired box 4 | 7q32.1 | rs6467136 | Intergenic | East Asian | 22158537 |
| *GIPR* | gastric inhibitory polypeptide receptor | 19q13.32 | rs8108269 | Downstream gene | European | 24509480 |
| *GLIS3* | GLIS family zinc finger 3 | 9p24.2 | rs7041847 | Intron | European | 24509480 |
|  |  |  | rs10814916 | Intron | East Asian, Singaporean Malay, South Asian | 22961080 |
|  |  |  | rs7041847 | Intron | East Asian | 22158537 |
| *GPSM1* | G-protein signaling modulator 1 | 9q34.3 | rs11787792 | Intron | East Asian | 23945395 |
| *GRB14* | Growth factor receptor-bound protein 14 | 2q24.3 | rs3923113 | Intergenic | European,South Asian | 24509480 |
| *GRK5* | G protein-coupled receptor kinase 5 | 10q26.11 | rs10886471 | Intron | East Asian, Singaporean Malay, South Asian | 22961080 |
| *HHEX* | Hematopoietically expressed homeobox | 10q23.33 | rs5015480 | Downstream gene | Chinese, European, East Asian | 22693455 |
|  |  |  | rs1111875 | Regulatory region | European, East Asian, Japanese | 23945395 |
| *HMG1L1, CTCFL, RBM38, PCK1* | High mobility group box 1 pseudogene 1; CCCTC-binding factor (zinc finger protein)-like; RNA binding motif protein 38; phosphoenolpyruvate carboxykinase 1 | 20q13.31 | rs328506 | Intergenic | Punjabi Sikh, South Asian, East Asian, European | 23300278 |
| *HMG20A* | High mobility group 20A | 15q24.3 | rs7178572 | Intron | European,South Asian | 24509480, 22693455, 21874001 |
|  |  |  | rs7119 | 3 prime UTR | Hispanic, European | 21490949 |
| *HMGA2* | High mobility group AT-hook 2 | 12q14.3 | rs2261181 | Intron | European | 24509480 |
|  |  |  | rs1531343 | Intron | European | 20581827 |
| *HNF1A* | HNF1 homeobox A | 12q24.31 | rs12427353 | Intron | European | 24509480 |
|  |  |  | rs7305618 | Intron | Hispanic, European | 21573907 |
|  |  |  | rs7957197 | Intron | European | 20581827 |
| *HNF1B* | HNF1 homeobox B | 17q12 | rs4430796 | Intron | European,East Asian, Singaporean Malay, South Asian | 24509480,23945395, 22961080, 20581827 |
| *HNF4A* | Hepatocyte nuclear factor 4, alpha | 20q13.12 | rs4812829 | Intron | European,South Asian | 24509480, 21874001 |
| *HUNK* | Hormonally up-regulated Neu-associated kinase | 21q22.11 | rs2833610 | Intron | Chinese, Malay, Asian Indian | 21490949 |
| *IGF2BP2* | Insulin-like growth factor 2 mRNA binding protein 2 | 3q27.2 | rs4402960 | Intron | European | 22693455, 24509480 |
|  |  |  | rs1470579 | Intron | East Asian,Punjabi Sikh, South Asian, East Asian, European | 23945395,23300278, 23300278, 23300278,23300278,23300278 |
|  |  |  | rs1374910 | Intron | Hispanic, European | 21573907 |
|  |  |  | rs4402960 | Intron | Japanese, European | 22693455, 24509480 |
|  |  |  | rs6769511 | Intron | Japanese, East Asian, European | 18711366 |
| *IL20RA* | Interleukin 20 receptor, alpha | 6q23.3 | rs6937795 | Downstream gene | European | 24509480 |
| *IRS1* | Insulin receptor substrate 1 | 2q36.3 | rs2943640 | Intergenic | European | 24509480 |
|  |  |  | rs7578326 | Intron | European | 20581827 |
| *JAZF1* | JAZF zinc finger 1 | 7p15.1 | rs849135 | Intron | European | 24509480 |
|  |  |  | rs849134 | Intron | European | 20581827 |
|  |  |  | rs864745 | Intron | European | 18372903 |
| *KCNJ11* | Potassium channel, inwardly rectifying subfamily J, member 11 | 11p15.1 | rs5215 | Missense | European | 24509480 |
| *KCNK16* | Potassium channel, two pore domain subfamily K, member 16 | 6p21.2 | rs1535500 | Missense | European ,East Asian | 24509480, 22158537 |
| *KCNQ1* | Potassium channel, voltage gated KQT-like subfamily Q, member 1 | 11p15.4 | rs163184 | Intron | European | 24509480 |
|  |  |  | rs2237897 | Intron | Mexican American,East Asian,South Asian, Japanese, European, African American,Native Hawaiian, Singaporean | 24390345 |
|  |  |  | rs8181588 | Intron | American Indian | 24101674 |
|  |  |  | rs2237892 | Intron | Japanese, East Asian, Singaporean Malay, South Asian,Hispanic, European | 23945395, 22961080 |
|  |  |  | rs163182 | Intron | Han Chinese | 21799836 |
|  |  |  | rs231362 | Non coding transcript exon | European | 20581827 |
|  |  |  | rs2237895 | Intron | Han Chinese | 20174558 |
| *KIF11* | Kinesin family member 11 | 10q23.33 | rs6583826 | Intergenic | Chinese, Malay, Asian Indian | 21490949 |
| *KLF14* | Kruppel-like factor 14 | 7q32.3 | rs972283 | Intergenic | European | 20581827 |
| *KLHDC5* | Kelch-like family member 42 | 12p11.22 | rs10842994 | Intergenic | European | 24509480 |
| *LINGO2* | Leucine rich repeat and Ig domain containing 2 | 9p21.1 | rs824248 | Intron | Mexican American,East Asian,South Asian, European, African American,Native Hawaiian, Singaporean | 24390345 |
| *LMNA* | Lamin A | 1q22 | rs4641 | Splice region | European, Danish | 17327460, 17327437 |
| *LPIN2* | Lipin 2 | 18p11.31 | rs10460009 | Intron | Chinese, Malay, Asian Indian | 21490949 |
| *LPP* | LIM domain containing preferred translocation partner in lipoma | 3q27.3 | rs6808574 | Intergenic | European | 24509480 |
| *LYPLAL1* | Lysophospholipase-like 1 | 1q41 | rs2820446 | Intergenic | European | 24509480 |
| *MAEA* | Macrophage erythroblast attacher | 4p16.3 | rs6815464 | Intron | East Asian | 23945395, 22158537 |
| *MAP3K1* | Mitogen-activated protein kinase kinase kinase 1, E3 ubiquitin protein ligase | 5q11.2 | rs10461617 | Intergenic | Indo-European, Dravidian, European | 23209189 |
| *MARCH1* | Membrane-associated ring finger (C3HC4) 1, E3 ubiquitin protein ligase | 4q32.3 | rs3792615 | Intron | Chinese, Malay, Asian Indian | 21490949 |
| *MC4R* | Melanocortin 4 receptor | 18q21.32 | rs12970134 | Intergenic | European | 24509480 |
| *MAEA* | Macrophage erythroblast attacher | 4p16.3 | rs7656416 | Intron | Japanese | 22456796 |
| *MIR129, LEP* | MicroRNA 129; leptin | 7q32.1 | rs791595 | Intron | East Asian | 23945395 |
| *MPHOSPH9* | M-phase phosphoprotein 9 | 12q24.31 | rs1727313 | 3 prime UTR | European | 24509480 |
| *MTNR1B* | Melatonin receptor 1B | 11q14.3 | rs10830963 | Intron | European | 24509480 |
|  |  |  | rs1387153 | Downstream gene | European | 20581827 |
| *NOTCH2, ADAM30* | Notch 2; ADAM metallopeptidase domain 30 | 1p12 | rs1092393 | Intron | European | 18372903 |
| *NXN* | Nucleoredoxin | 17p13.3 | rs623323 | Downstream gene | Punjabi Sikh, South Asian, East Asian, European | 23300278 |
| *PALM2, AKAP2* | Paralemmin 2; A kinase (PRKA) anchor protein 2 | 9q31.3 | rs1327796 | Intron | Japanese | 22456796 |
| *PCBD2* | Pterin-4 alpha-carbinolamine dehydratase/dimerization cofactor of hepatocyte nuclear factor 1 alpha (TCF1) 2 | 5q31.1 | rs319598 | Upstream gene | European | 24509480 |
| *PCNXL2* | Pecanex-like 2 | 1q42.2 | rs12027542 | Intron;Intron | Chinese, Malay, Asian Indian | 21490949 |
| *PEPD* | Peptidase D | 19q13.11 | rs3786897 | Intron | East Asian | 22158537 |
| *PEX5L* | Peroxisomal biogenesis factor 5-like | 3q26.33 | rs7630877 | Intron | Chinese, Malay, Asian Indian | 21490949 |
| *PLEKHA1* | Pleckstrin homology domain containing, family A (phosphoinositide binding specific) member 1 | 10q26.13 | rs10510110 | 3 prime UTR | European | 24509480 |
| *PLS1* | Plastin 1 | 3q23 | rs3773506 | 3 prime UTR | Chinese, Malay, Asian Indian | 21490949 |
| *POU5F1,TCF19* | POU class 5 homeobox 1 ; transcription factor 19 | 6p21.33 | rs3132524 | Intron | European | 24509480 |
| *PPARG* | Peroxisome proliferator-activated receptor gamma | 3p25.2 | rs1801282 | Missense | European | 17463246 |
|  |  |  | rs13081389 | Intergenic | European | 20581827 |
| *PRC1* | Protein regulator of cytokinesis 1 | 15q26.1 | rs12899811 | Intron | European | 24509480 |
|  |  |  | rs8042680 | Intron | European | 20581827 |
| *PROX1* | Prospero homeobox 1 | 1q32.3 | rs2075423 | Intron | European | 24509480 |
| *PSMD6* | Proteasome (prosome, macropain) 26S subunit, non-ATPase, 6 | 3p14.1 | rs831571 | Intergenic | East Asian | 22158537 |
| *PTEN* | Phosphatase and tensin homolog | 10q23.31 | rs10788575 | Intergenic | European | 24509480 |
| *PTPRD* | Protein tyrosine phosphatase, receptor type, D | 9p23 | rs649891 | Intron | Mexican American | 21647700 |
|  |  |  | rs17584499 | Intron | Han Chinese | 20174558 |
| *RASGRP1* | RAS guanyl releasing protein 1 (calcium and DAG-regulated) | 15q14 | rs7403531 | Intron | East Asian, Singaporean Malay, South Asian | 22961080 |
| *RBM43, RND3* | RNA binding motif protein 43; Rho family GTPase 3 | 2q23.3 | rs7560163 | Intergenic | African American | 22238593 |
| *RBMS1, ITGB6* | RNA binding motif, single stranded interacting protein 1 ; integrin beta 6 | 2q24.2 | rs7593730 | Intron | European | 20418489 |
| *RHOU* | Ras homolog family member U | 1q42.13 | rs6426514 | 3 prime UTR | Punjabi Sikh, South Asian, East Asian, European | 23300278 |
| *RNF6* | Ring finger protein (C3H2C3 type) 6 | 13q12.13 | rs10507349 | Intron | European | 24509480 |
| *SGCG, SACS* | Sarcoglycan, gamma (35kDa dystrophin-associated glycoprotein); sacsin molecular chaperone | 13q12.12 | rs9552911 | Intron | Punjabi Sikh, South Asian, East Asian, European | 23300278 |
| *SLC16A11, SLC16A13* | Solute carrier family 16, member 11; solute carrier family 16 (monocarboxylic acid transporters), member 13 | 17p13.1 | rs75493593 | Missense | Mexican American,East Asian,South Asian, European, African American,Native Hawaiian, Singaporean | 24390345 |
| *SLC16A13* | Solute carrier family 16 (monocarboxylic acid transporters), member 13 | 17p13.1 | rs312457 | Intron | East Asian | 23945395 |
| *SLC30A8* | Solute carrier family 30 (zinc transporter), member 8 | 8q24.11 | rs13266634 | Missense | East Asian | 19401414 |
|  |  |  | rs3802177 | 3 prime UTR | European | 22693455, 20581820 |
|  |  |  | rs13266634 | Missense | Japanese, European | 19056611, 17463246, 17463248, 17460697, 17463249, 17293876 |
| *SLC44A3, F3* | Solute carrier family 44, member 3 ; coagulation factor III (thromboplastin, tissue factor) | 1p21.3 | rs7542900 | Intergenic | African American | 22238593 |
| *SPRY2* | Sprouty homolog 2 | 13q31.1 | rs1359790 | Intergenic | Chinese, European, East Asian | 24509480, 20862305 |
| *SRR* | Serine racemase | 17p13.3 | rs391300 | Intron | Han Chinese | 20174558 |
| *SSR1,RREB1* | Ras responsive element binding protein 1 | 6p24.3 | rs9502570 | Intergenic | European | 24509480 |
| *ST6GAL1* | ST6 beta-galactosamide alpha-2,6-sialyltranferase 1 | 3q27.3 | rs16861329 | Intron | European, South Asian | 24509480, 21874001 |
| *SYK* | Spleen tyrosine kinase | 9q22.2 | rs10993738 | Intron | Japanese | 21874001 |
| *SYN2, PPARG* | Synapsin II; peroxisome proliferator-activated receptor gamma | 3p25.2 | rs17036101 | Regulatory region | European | 18372903 |
| *TCERG1L* | Transcription elongation regulator 1-like | 10q26.3 | rs10741243 | Intron | Chinese, Malay, Asian Indian | 21490949 |
| *TCF7L2* | Transcription factor 7-like 2 (T-cell specific, HMG-box) | 10q25.2 | rs7903146 | Intron | Mexican American,East Asian, Indo-European, Dravidian,South Asian, European,Punjabi Sikh, African American, Japanese, Finland founder,Ashkenazi Jewish Native Hawaiian,Singaporean | 24509480, 24390345, 23945395, 23300278, 23209189, 22693455, 22101970, 20581827, 19401414, 19056611, 18372903, 1766838217668382, 17554300, 17463246, 17463248, 17460697, 17463249, 17293876, 20581827, 22158537, 20581827, 24509480, 21490949 |
|  |  |  | rs4506565 | Intron | European | 17554300 |
| *TGFBR3* | Transforming growth factor, beta receptor III | 1p22.1 | rs11165354 | Intron | Indo-European, Dravidian, European | 23209189 |
| *THADA* | Thyroid adenoma associated | 2p21 | rs7578597 | Missense | European | 18372903 |
| *TLE1* | Transducin-like enhancer of split 1 | 9q21.32 | rs2796441 | Intron | European | 24509480 |
| *TLE4* | Transducin-like enhancer of split 4 | 9q21.31 | rs17791513 | Intergenic | European | 24509480 |
| *TMEM154* | Transmembrane protein 154 | 4q31.3 | rs6813195 | Intergenic | European | 24509480 |
| *TMEM163* | Transmembrane protein 163 | 2q21.3 | rs6723108 | Upstream gene | Indo-European, Dravidian, European | 23209189 |
| *TMEM18* | Transmembrane protein 18 | 2p25.3 | rs10190052 | Intergenic | European | 24509480 |
| *TMEM45B, BARX2* | Transmembrane protein 45B; BARX homeobox 2 | 11q24.3 | rs7107217 | Intron | African American | 22238593 |
| *TMEM75* | TMEM75 transmembrane protein 75 | 8q24.21 | rs1561927 | Intron | European | 24509480 |
| *TP53INP1* | Tumor protein p53 inducible nuclear protein 1 | 8q22.1 | rs7845219 | Intron | European | 24509480 |
|  |  |  | rs896854 | Intron | European | 20581827 |
| *TSPAN8,LGR5* | Tetraspanin 8 | 12q21.1 | rs4760790 | Intron | European | 20581827 |
|  |  |  | rs7961581 | Intron | European | 18372903 |
| *UBE2E2* | Ubiquitin-conjugating enzyme E2E 2 | 3p24.3 | rs7612463 | Intron | European | 24509480 |
|  |  |  | rs6780569 | Intergenic | East Asian | 23945395 |
| *VEGFA* | Vascular endothelial growth factor A | 6p21.1 | rs9472138 | Intergenic | European | 24509480, 18372903 |
| *VPS26A* | Vacuolar protein sorting 26 homolog A | 10q22.1 | rs1802295 | 3 prime UTR | South Asian | 21874001 |
| *WFS1* | Wolfram syndrome 1 | 4p16.1 | rs4458523 | Intron | European | 24509480 |
|  |  |  | rs1801214 | Missense | European | 20581827 |
|  |  |  | rs4689388 | Upstream gene | European | 19734900 |
| *WISP1* | WNT1 inducible signaling pathway protein 1 | 8q24.22 | rs4527850 | Regulatory region | Punjabi Sikh, South Asian, East Asian, European | 23300278 |
| *WWOX* | WW domain containing oxidoreductase | 16q23.2 | rs17797882 | Regulatory region | East Asian | 22158537 |
| *ZBED3* | Zinc finger, BED domain containing 3 | 5q13.3 | rs4457053 | Intron | European | 20581827 |
| *ZFAND3* | Zinc finger, AN1-type domain 3 | 6p21.2 | rs9470794 | Intron | East Asian | 22158537 |
| *ZFAND6* | Zinc finger, AN1-type domain 6 | 15q25.1 | rs11634397 | Downstream gene | European | 20581827 |
| *ZMIZ1* | Zinc finger, MIZ-type containing 1 | 10q22.3 | rs12571751 | Intronic | European | 24509480 |
| *ZPLD1* | Zona pellucida-like domain containing 1 | 3q12.3 | rs2063640 | Downstream gene | Chinese, Malay, Asian Indian | 21490949 |

SNP, single nucleotide polymorphism; UTR, untranslated region
